# Intronic Non-CG DNA hydroxymethylation and alternative mRNA splicing in honey bees

**DOI:** 10.1186/1471-2164-14-666

**Published:** 2013-09-30

**Authors:** Pablo Cingolani, Xiaoyi Cao, Radhika S Khetani, Chieh-Chun Chen, Melissa Coon, Alya’a Sammak, Aliccia Bollig-Fischer, Susan Land, Yun Huang, Matthew E Hudson, Mark D Garfinkel, Sheng Zhong, Gene E Robinson, Douglas M Ruden

**Affiliations:** 1 Department of Obstetrics and Gynecology, Wayne State University, Detroit, MI 48201, USA; 2 School of Computer Science & Genome Quebec Innovation Centre, McGill University, Montreal, QC, Canada; 3 Institute for Genomic Biology, University of Illinois, Urbana, IL 61801, USA; 4 Department of Crop Sciences, University of Illinois, Urbana, IL 61801, USA; 5 Department of Oncology, Wayne State University, Detroit, MI 48201, USA; 6 La Jolla Institute for Allergy & Immunology, La Jolla, CA 92037, USA; 7 Department of Biological Sciences, University of Alabama in Huntsville, Huntsville, AL, USA; 8 Department of Entomology, University of Illinois, Urbana, IL 61801, USA; 9 Neuroscience Program, University of Illinois, Urbana, IL 61801, USA; 10 Institute of Environmental Health Sciences, Wayne State University, Detroit, MI 48201, USA

**Keywords:** Honey Bees, DNA methylation, DNA hydroxymethylation, Epigenetics

## Abstract

**Background:**

Previous whole-genome shotgun bisulfite sequencing experiments showed that DNA cytosine methylation in the honey bee (*Apis mellifera*) is almost exclusively at CG dinucleotides in exons. However, the most commonly used method, bisulfite sequencing, cannot distinguish 5-methylcytosine from 5-hydroxymethylcytosine, an oxidized form of 5-methylcytosine that is catalyzed by the TET family of dioxygenases. Furthermore, some analysis software programs under-represent non-CG DNA methylation and hydryoxymethylation for a variety of reasons. Therefore, we used an unbiased analysis of bisulfite sequencing data combined with molecular and bioinformatics approaches to distinguish 5-methylcytosine from 5-hydroxymethylcytosine. By doing this, we have performed the first whole genome analyses of DNA modifications at non-CG sites in honey bees and correlated the effects of these DNA modifications on gene expression and alternative mRNA splicing.

**Results:**

We confirmed, using unbiased analyses of whole-genome shotgun bisulfite sequencing (BS-seq) data, with both new data and published data, the previous finding that CG DNA methylation is enriched in exons in honey bees. However, we also found evidence that cytosine methylation and hydroxymethylation at non-CG sites is enriched in introns. Using antibodies against 5-hydroxmethylcytosine, we confirmed that DNA hydroxymethylation at non-CG sites is enriched in introns. Additionally, using a new technique, Pvu-seq (which employs the enzyme PvuRts1l to digest DNA at 5-hydroxymethylcytosine sites followed by next-generation DNA sequencing), we further confirmed that hydroxymethylation is enriched in introns at non-CG sites.

**Conclusions:**

Cytosine hydroxymethylation at non-CG sites might have more functional significance than previously appreciated, and in honey bees these modifications might be related to the regulation of alternative mRNA splicing by defining the locations of the introns.

## Background

Methylation of DNA is increasingly appreciated as a potent way to regulate gene expression, but a comprehensive understanding of the diversity of methylation mechanisms has not yet been achieved. For example, methylation that does not occur at cytosine-guanosine dinucleotide sequences (non-CG methylation) is an underappreciated and poorly understood form of epigenetic regulation. While rare in mammalian somatic cells, non‒CG methylation occurs on 20‒40% of the cytosines in human embryonic stem cells (hESCs) [1,2], and is thought to be involved in pluripotency [1]. A recent comparative analysis of DNA methylation across hESC lines found that heavily methylated non‒CG sites are strongly conserved, especially within the motif TA^m^CAG at 3’ splice junctions [2], suggesting a role in splicing or alternative splicing of mRNA transcripts. Also, CTCF induced RNA polymerase II pausing was shown to link alternative mRNA splicing to DNA methylation at a CTCF binding site in genomic DNA encoding an intron [3]. Recently it was shown that RNA interference knockdown of DNA methyltransferase 3 (Dnmt3) affects gene alternative splicing in the honey bee [4].

The honey bee (*Apis mellifera*) is emerging as a new model to study effects of methylation on genome function because, unlike *Drosophila melanogaster*, it possesses a fully functioning methylation system [5-7]. Three studies [6,8,9] have reported that honey bees have CG methylation primarily at exon coding regions, and we have confirmed these studies here. These three studies have also reported that honey bees have little, if any, non-CG methylation [6,8,9]. However, since all three of the previous studies’ experimental design filtered out much of the non-CG methylation ( [6,8,9] and personal communication; see Acknowledgements), it is still an open question as to whether there are significant amounts of non-CG methylation in bees. The are several reasons for filtering out non-CG methylation: (1) non-CG methylation is much less abundant than CG methylation in mammals; (2) there are several times more non-CG sequences (i.e., CA, CT, and CC) than CG sequences, and focusing analyses on CG methylation is simpler; (3) non-CG methylation is often in less complex regions of the genome, such as introns, and is therefore difficult to map with short-read next-generation sequencing technology; and (4) since bisulfite works less well on double-stranded DNA than single-stranded DNA, less complex regions might form snap-back structures that are resistant to bisulfite conversion.

Hydroxymethylation of DNA is a newly discovered form of epigenetic regulation. It has been found recently in embryonic stem cells (ESC) and in the brains of mammals [10,11]. In mammals, TET proteins have been shown to be dioxygenases that convert ^m^C to 5-hydroxymethylcytosine (^hm^C). Honey bees have a TET ortholog [10,12], but ^hm^C has not yet been reported. Genome-wide wide mapping in mouse ESCs has revealed that ^hm^C is enriched at the start sites of genes whose promoters bear histone 3 lysine 27 trimethylation (H3K27me3) and histone 3 lysine 4 trimethylation (H3K4me3) marks [13-18]. In human ESCs, this dual mark is derived from separable subpopulations of self-renewing and lineage-biased ESCs within the heterogeneous unfractionated ESC population [19].

The most common chemical approach to study DNA methylation is treating single-stranded DNA with bisulfite, but bisulfite cannot distinguish ^m^C from ^hm^C because both base-pair as cytosine after bisulfite treatment. The modification ^m^C mostly remains in this form after bisulfite treatment, whereas ^hm^C is converted to cytosine methylene sulfonate (CMS) after bisulfite treatment, which has the same hydrogen bond donor and acceptor configuration as cytosine for base pairing to guanine [10]. In this paper, in addition to whole-genome shotgun bisulfite sequencing of honey bee head DNA, we developed new biochemical and bioinformatics tools to analyze non-CG methylation and hydroxymethylation. We sharpened our analyses by comparing bees endemic to North America that are derived from a mixture of European subspecies of *Apis mellifera* (“EHB”) with bees derived from the African subspecies *Apis mellifera scutallata*, introduced to South America in the last century (“AHB”). EHB and AHB are attractive candidates for comparative molecular analysis because they differ in many physiological and behavioral traits, including aggression. Differences in methylation between EHB and AHB that are reported here may be related to these physiological and behavioral differences, and could motivate further studies, beyond the scope of the present paper. We show that non-CG methylation and hydroxymethylation are both enriched in introns, and cytosine modification at splice junctions might help regulate alternative splicing and gene expression.

## Results and discussion

We asked whether non-CG methylation and hydroxymethylation occurs in honey bees using unbiased approaches that do not filter out non-CG methylation. We compared AHB and EHB mainly to demonstrate the robustness of our experimental and bioinformatics approaches. To standardize our comparisons, we analyzed head methylomes only from AHB and EHB “guard” bees, a specialized group of individuals that patrol the hive entrance and respond first to a threat to their colony. Most of the tissues in the bee head consist of brain.

### Whole-genome shotgun bisulfite sequencing validates that CG methylation is primarily located in the exons

We performed whole-genome shotgun sequencing of bisulfite modified DNA (BS-Seq) from AHB and EHB heads and obtained over 20× coverage of both genomes (Table 1; Additional file 1: Figure S1; see Methods). All four of the honey bee BS-Seq studies conducted to date — three published studies [6,8,9], and our experiments reported here — identified ~5-10× more CG methylation in exons than in introns. In the present study, there was ~6% CG methylation in exons and ~1% in introns and intergenic regions in EHB, compared with ~3% and ~0.3%, respectively in AHB (Table 2). Similarly, our re-analysis of the data from ref. [6] identified ~8% CG methylation in exons and ~0.3% methylation in introns in EHB (AHB was not studied; Additional file 2: Table S1). Only 15% (AHB) to 21% (EHB) of the CG methylation is symmetrically methylated in honey bees (Table 1), which is lower than what is observed in mammals (over 90%). Our analysis methods, which we call BS-Miner (See Methods), are sensitive to non-CG sites and identify hemi-methylated DNA using algorithms analogous to those by which heterozygous DNA sequences are identified in whole-genome sequences [20]. Recently, a BS-seq analysis tool called Bismark was developed that does not filter out non-CG methylation [21]. The amount of CHH and CHG methylation identified in the ref. [6] dataset by Bismark was approximately the same as the amount of CG methylation (516,148 versus 540,208, Additional file 2: Table S2), which is consistent with our analyses of our AHB and EHB datasets that show much more CHH and CHG methylation than previously reported.

**Table 1:** Differential cytosine DNA methylation between European (EHB) and Africanized (AHB) honey bees in CG, CHG, and CHH genomic contexts (H = A, T, or C)

Total*		**Methylated in EHB (%)**	**Methylated in AHB (%)**	**Symmetrically methylated (%)**
CG	10,030,209 (15%)	253,041 (2.5%)	94,248 (0.9%)	54,120 (21%, EHB), 14,454 (15%, AHB)
CHG	8,673,113 (14%)	80,295 (0.9%)	24,834 (0.3%)	423 (0.53%, EHB), 106 (0.43%, AHB)
CHH	45,072,611 (71%)	1,258,515 (2.8%)	519,318 (1.2%)	0%
Total	63,775,933 (100%)	1,591,851 (2.5%)	638,400 (1.1%)	54,543 (3.4%, EHB), 14,560 (2.3% AHB)

*The total number of cytosines represent both the uniquely mapped and the non-uniquely mapped cytosines (from [6]).

**Table 2:** DNA methylation at CpG sites in AHB and EHB

**AHB CpG methylation by genetic region**			
**Type (alphabetical order)**	**Analyzed**	**Methylated**	**Methylated %**
Cds	1,273,706	40,288	3.16%
Downstream	2,086,041	37,798	1.81%
Exon	1,273,706	40,288	**3.16%**
Gene	7,105,672	62,011	0.87%
Intergenic	9,767,537	35,409	0.36%
Intron	5,887,038	22,463	**0.38%**
SpliceSiteAcceptor	14	1	7.14%
SpliceSiteDonor	2,257	81	3.59%
Transcript	7,806,591	64,990	0.83%
Upstream	2,294,855	38,169	1.66%
**EHB CpG methylation by genetic region**			
**Type (alphabetical order)**	**Analyzed**	**Methylated**	**Methylated %**
Cds	1,318,533	82,558	6.26%
Downstream	2,255,737	89,512	3.97%
Exon	1,318,533	82,558	**6.26%**
Gene	7,555,968	149,371	1.98%
Intergenic	10,471,326	111,682	1.07%
Intron	6,293,493	68,460	**1.09%**
SpliceSiteAcceptor	18	1	5.56%
SpliceSiteDonor	2,504	215	8.59%
Transcript	8,297,751	158,204	1.91%
Upstream	2,468,142	87,960	3.56%

### Whole-genome shotgun bisulfite sequencing identifies non-CG modifications that are enriched in introns

As in the previous studies [6,8,9], we determined that CG methylation is primarily in the exons. However, a second finding from our analyses of both our data and the data from ref. [6] is relatively high levels of non-CG modifications (i.e., either ^m^C or ^hm^C) in bee heads. Surprisingly, our methods detected about 5-fold more CHH modifications (H = C, A, T) than CG methylation in both AHB and EHB DNA (Table 1). As with the CG methylation, we also saw more than twice as many non-CG modifications in EHB than AHB heads. About 2.5% of the total number of CHH sequences was modified in EHB and about 1.1% in AHB (Table 1).

In order to validate our finding of high levels of non-CG modifications, we reanalyzed previously published honey bee data from ref. [6] with our methods and again found that the amount of non-CG modifications exceeded the amount of CG methylation (Additional file 2: Table S1). The differences in the amounts of non-CG modifications in our data compared with ref. [6] might be caused by bisulfite treatment conditions (we did a single treatment and they did multiple treatments) or differences in strains of bees used in the two studies. We constructed the Illumina libraries using the identical protocol as ref. [6] which used unmethylated oligonucleotides, followed by amplification of only bisulfite converted oligonucleotides, to ensure that only fully bisulfite converted DNA is incorporated into the libraries (see Methods).

We also validated our data analysis pipelines by using two recent independently developed bisulfite methylation analysis program (BS-Map and Bismark) [21-23] on the data from ref. [6] and were again able to identify non-CG methylation sites (Additional file 2: Table S2 and Additional file 1: Figure S2). These independent algorithms also found more non-CG than CG methylation, consistent with our findings.

In contrast to CG methylation, CHH modifications were highest in introns (~4% and ~2% in EHB and AHB) and lowest in exons (~2% and ~0.8% in EHB and AHB) (Table 3). Consistent with this, our re-analysis of the EHB data from ref. [6] identified ~4 times more CHH modifications in introns than exons (2% versus 0.5%; Additional file 2: Table S1).

**Table 3:** DNA methylation at CHH sites in AHB and EHB

**AHB CHH modifications by genetic region**			
**Type (alphabetical order)**	**Analyzed**	**Methylated**	**Methylated %**
Cds	3,316,927	26,207	0.79%
Downstream	5,075,583	79,404	1.56%
Exon	3,316,927	26,207	**0.79%**
Gene	12,684,330	212,020	1.67%
Intergenic	16,317,387	325,139	1.99%
Intron	9,480,307	186,385	**1.97%**
SpliceSiteAcceptor	14,774	87	0.59%
SpliceSiteDonor	26,957	121	0.45%
Transcript	13,795,281	234,094	1.70%
Upstream	5,213,214	74,112	1.42%
**EHB CHH modifications by genetic region**			
**Type (alphabetical order)**	**Analyzed**	**Methylated**	**Methylated %**
Cds	3,648,122	85,111	2.33%
Downstream	5,967,723	216,950	3.64%
Exon	3,648,122	85,111	**2.33%**
Gene	14,525,454	521,600	3.59%
Intergenic	19,011,050	773,971	4.07%
Intron	10,997,021	438,582	**3.99%**
SpliceSiteAcceptor	17,187	538	3.13%
SpliceSiteDonor	34,121	620	1.82%
Transcript	15,784,142	570,790	3.62%
Upstream	6,096,005	211,100	3.46%

We also detected CHG modifications at levels lower than both CG and CHH (~1.1% and ~0.3% in EHB and AHB, Table 4). There was only about one-seventh as many CHG modifications as CHH modifications, and very few of the CHG modifications were symmetrical (~3.4% in EHB and ~2.3% in AHB, Table 2). CHG modifications were more uniform across the genome than CG methylation or CHH modifications (Table 2). Coverage for individual chromosomes (Additional file 1: Figure S3) demonstrates that there were no significant biases toward any portion of the genome in the sequencing procedure.

**Table 4:** DNA methylation at CHG sites in AHB and EHB

**AHB CHG methylation by genetic region**			
**Type (alphabetical order)**	**Analyzed**	**Methylated**	**Methylated %**
Cds	1,002,706	3,493	0.35%
Downstream	1,119,219	4,559	0.41%
Exon	1,002,706	3,493	0.35%
Gene	3,188,727	11,619	0.36%
Intergenic	3,777,833	14,009	0.37%
Intron	2,225,532	8,217	0.37%
SpliceSiteAcceptor	16,858	43	0.26%
SpliceSiteDonor	4,407	15	0.34%
Transcript	3,470,589	12,577	0.36%
Upstream	1,189,498	4,526	0.38%
**EHB CHG methylation by genetic region**			
**Type (alphabetical order)**	**Analyzed**	**Methylated**	**Methylated %**
Cds	1,059,063	11,898	1.12%
Downstream	1,242,115	16,772	1.35%
Exon	1,059,063	11,898	1.12%
Gene	3,419,998	37,559	1.10%
Intergenic	4,099,352	45,117	1.10%
Intron	2,401,495	25,966	1.08%
SpliceSiteAcceptor	18,474	257	1.39%
SpliceSiteDonor	5,072	57	1.12%
Transcript	3,718,898	40,695	1.09%
Upstream	1,308,187	16,511	1.26%

Overall, EHB had almost 2.5× more modified Cs than did AHB (1,591,851 versus 638,400). We also observed ~3-4-fold more CG methylation than in the previous three studies: we detected 253,041 methylated CGs in EHB, compared with 80,000-90,000 in the previous 3 studies (Table 2) [6,8,9]. This appears to be due to higher sensitivity of the analysis program used; as stated above, using our methods on data from ref. [6] identified 334,949 methylated CGs (Additional file 2: Table S1), which is even more than we identified in our data. The significance of EHB having 2.5× more modified Cs than AHB is not known.

### Bees have 5-hydroxymethylcytosine

BS-Seq cannot distinguish ^m^C from ^hm^C because both base pair as C after BS treatment [10]. We used highly sensitive anti-CMS antibodies [13] to determine the levels of ^hm^C in the heads and bodies of AHB and EHB. Consistent with the BS-Seq results, we found comparable and statistically indistinguishable levels of ^hm^C in EHB and AHB heads (15.2 pmol/μg and 13.5 pmol/μg of genomic DNA) (Additional file 1: Figure S4). For bodies, there was significantly more ^hm^C in EHB than AHB (25.7 pmol/μg and 19.3 pmol/μg) (p < 0.05, 2-tailed t-test, Additional file 1: Figure S4).

### The 5-hydroxymethylcytosine in bees is enriched in introns

To distinguish ^m^C from ^hm^C, we immunoprecipitated honey bee head DNA with antibodies against 5-hydroxymethylcytosine (HMeDIP). The immunoprecipitated DNA was then sequenced by next-generation DNA sequencing (HMeDIP-seq). Compared to previous findings that ^m^C is found primarily in exons, we found that most of the ^hm^C DNA is present in introns (Table 5), where most of the non-CG modifications are also present. This leads to the speculation that ^hm^C is enriched in non-CG sites, and that many of the non-CG modifications that are detected by whole genome bisulfite sequencing are actually ^hm^C, since bisulfite cannot distinguish ^m^C from ^hm^C.

**Table 5:** Pvu-seq and HMeDIP-seq results show that cytosine hydroxymethylation is enriched in introns in the honey bee genome

**Pvu-seq AHB**		
**Type (alphabetical order)**	**Count**	**Percent**
Downstream	114,800	10.50%
Exon	138,670	12.68%
Intergenic	367,126	33.58%
Intragenic	1,542	0.14%
Intron	245,458	**22.45%**
Splice_Site_Acceptor	46,519	4.25%
Splice_Site_Donor	46,039	4.21%
Upstream	133,265	12.19%
**Pvu-seq EHB**		
**Type (alphabetical order)**	**Count**	**Percent**
Downstream	277,098	10.80%
Exon	312,968	12.20%
Intergenic	852,968	33.25%
Intragenic	3,332	0.13%
Intron	592,529	**23.10%**
Splice_Site_Acceptor	107,318	4.18%
Splice_Site_Donor	108,663	4.24%
Upstream	310,213	12.09%
**HMeDIP-seq AHB**		
**Type (alphabetical order)**	**Count**	**Percent**
Downstream	2,255	10.98%
Gene	2,537	12.35%
Intergenic	6,312	30.72%
Intron	4,487	**21.84%**
None	2	0.01%
Splice_Site_Acceptor	1,343	6.54%
Splice_Site_Donor	1,373	6.68%
Upstream	2,238	10.89%

### Pvu-seq validates the location of ^hm^C in introns

**Figure 1:**
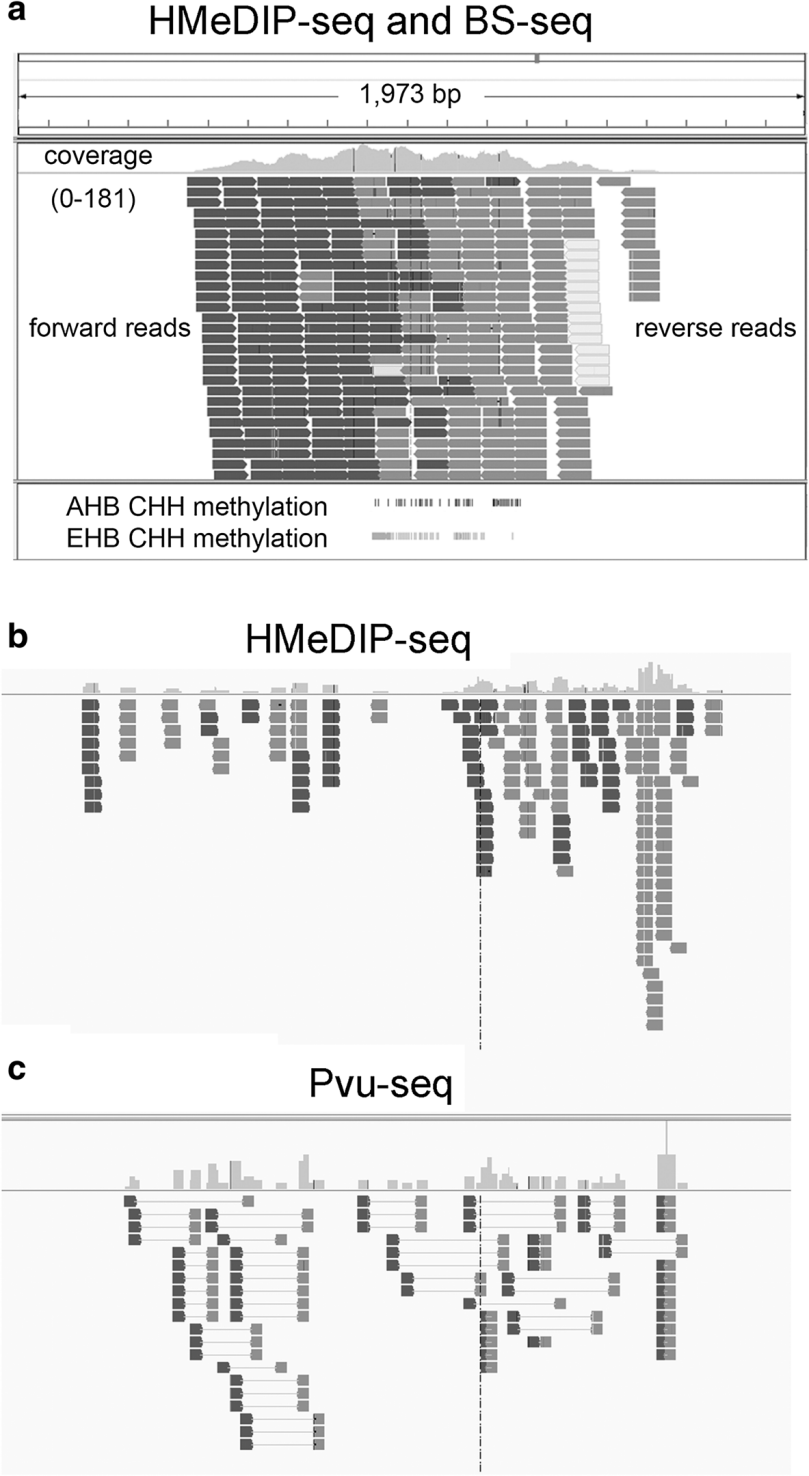
**Validation of CHH modifications based on MeDIP-Seq. a**, A large MeDIP peak in Africanized honey bees (AHB) that has a large amount of CHH modifications but no CG methylation in both European (EHB) and AHB. Coverage (0-181) represents the sequence coverage of mDIP-Seq fragments (the gray line is 0 and the top of the peak is 181). Forward reads are shown in black and reverse reads are shown in gray. The white boxes show reverse reads that did not have forward reads that could be aligned uniquely to the genome. The bottom portion shows the CHH modifications in AHB and EHB. **b**, HMeDIP-Seq analysis of a region of the genome that has a large peak of immunoprecipitated DNA. The histogram on the top shows the relative number of RNA-Seq fragments that align to the indicated region of the genome. Forward sequences are shown in red and reverse sequences in blue. **c**, Pvu-Seq analysis of the same region of the genome. Notice the good concordance between the two analyses.

To validate the ^hm^C findings, we developed a new technique that we call Pvu-Seq. This technique involves digesting the DNA isolated from AHB and EHB heads with the Type 2 restriction endonuclease, PvuRts1I, which cuts near single hydroxymethylcytosine sites [24-26]. The distances between the cleavage sites and the modified cytosine are fixed within a narrow range, with the majority being 11-13 nucleotides away in the top strand and 9-10 nucleotides away in the bottom strand [24,25]. There was an excellent correlation between hmDIP-Seq and Pvu-Seq data; over 89% of the HMeDIP-Seq peaks were also represented by Pvu-Seq peaks. An example of an HMeDIP-Seq peak correlating with a Pvu-Seq reads in AHB is shown in Figure 1b and c. These results confirm the findings made with other techniques and indicate that Pvu-Seq is a valid technique for mapping ^hm^C sites in the AHB and EHB genomes.

### There are more non-CG modifications in genes with a low CG content

**Figure 2:**
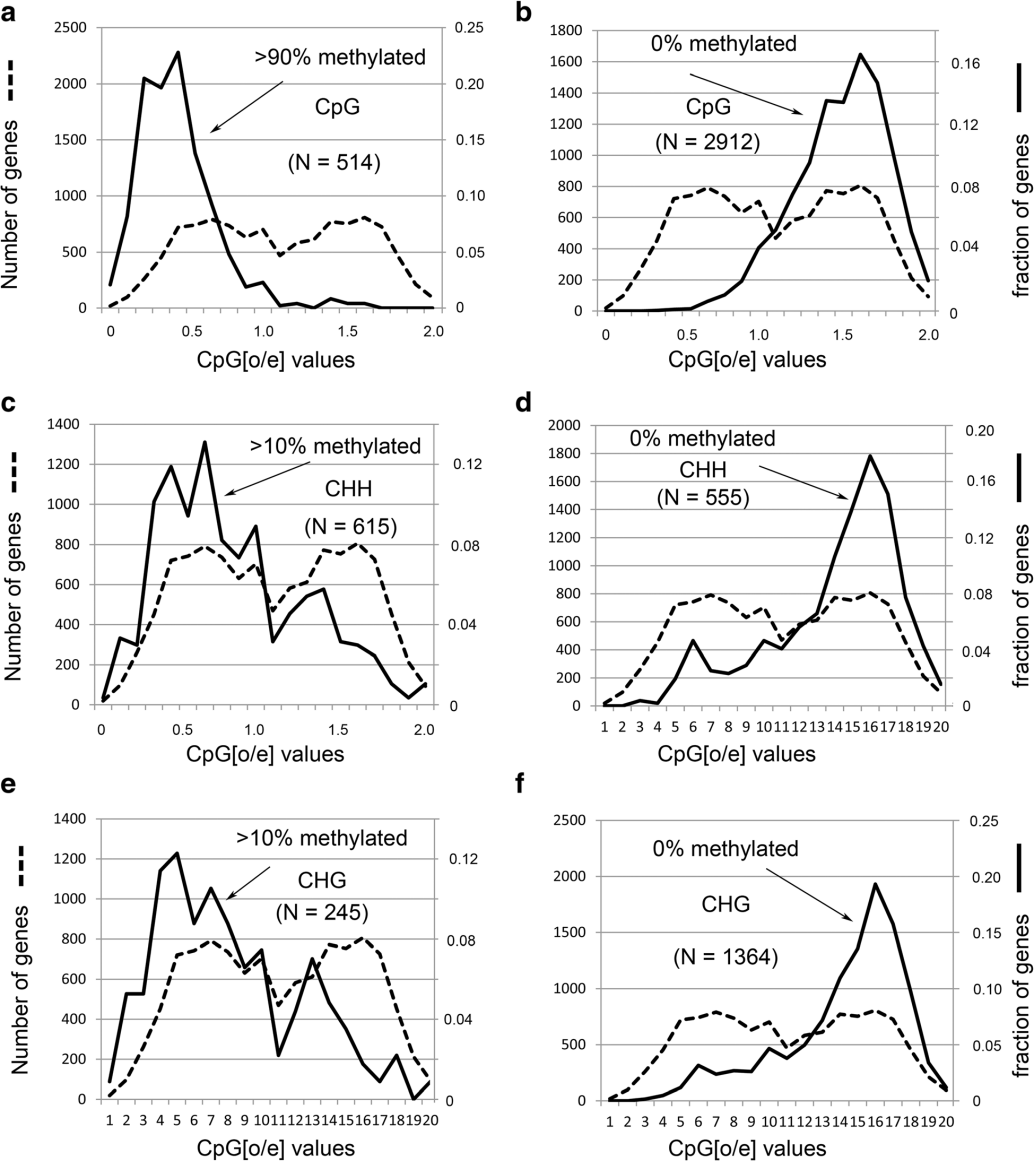
**Cytosine modifications in introns are predominantly in the low-CpG content genes. a**, plot of EHB genes (N = 514) with >90% methylation at CpG sites. The dashed line shows the bimodal distribution of bee genes with low o/e and high o/e (observed/expected) CpG ratios. The number of genes in the bimodal o/e plot is shown on the left Y-axis. The percentage of genes with >90% methylation in each region of the bimodal o/e plot is shown on the right Y-axis. **b**, plot of EHB genes (N = 2912) with zero percent CpG methylation. Since there are a large number of genes in all three classes with zero percent methylation, we restricted our analyses to genes with multiple motifs as follows: for CpG, there are 2912 genes with at least 10 total CpGs in the exons with 0% methylation. **c**, plot of EHB genes (N = 615) with >10% methylation at CHH sites in introns. **d**, plot of EHB genes (N = 555) with zero percent methylation at CHH sites in introns. For CHH, there are 555 genes with zero percent methylation in introns that have at least 400 total CHHs in the introns. **e**, plot of EHB genes (N = 245) with >10% methylation at CHG sites in introns. **f**, plot of EHB genes (N = 1364) with zero percent methylation at CHG sites in introns. For CHG, there are 1364 genes with zero percent methylation with at least 100 total CHGs in the introns.

Previous analyses in honey bees have shown that there are two classes of genes with respect to CG methylation: one has a low observed over expected (o/e) CG ratio (i.e., low CG content), is highly methylated, and is enriched in housekeeping genes, and a second has a high o/e ratio (i.e., high CG content), is unmethylated, and is enriched in caste-specific and developmental genes (Figure 2, dashed lines; Additional file 1: Figure S5) [6,8,27-29]. Although non-CG sequences have a unimodal distribution (Additional file 1: Figure S6), rather than a bimodal distribution in the genome, non-CG modifications in introns surprisingly were found primarily in genes with a low o/e CG ratio (Figure 2). Genes with greater than 10% non-CG modifications, such as ^m^C and ^hm^C, in introns are primarily in low o/e CG genes (i.e., the left peak in the o/e CG ratio plot, dashed lines; Figure 2), whereas genes with zero percent non-CG modifications in introns are in high o/e CG genes (i.e., the right peak in the o/e CG ratio plot, dashed lines; Figure 2). Therefore, the pattern of methylation is dependent on the CG dinucleotide content and not the CHH or CHG trinucleotide content. We speculate that this might indicate that CG methylation is somehow linked to non-CG modifications, possibly via interactions between the maintenance DNA methyltransferase, Dnmt1, and the *de novo* enzyme, Dnmt3, both of which are present in bees [5], and the bee TET protein. In contrast to Dnmt1, which has almost exclusive specificity for CG sites (however, see ref. [30]), Dnmt3 is responsible for most non-CG methylation in hESC [31]; this has not been examined in bees.

### CHH modifications are enriched in the introns of genes that regulate transcription

**Figure 3:**
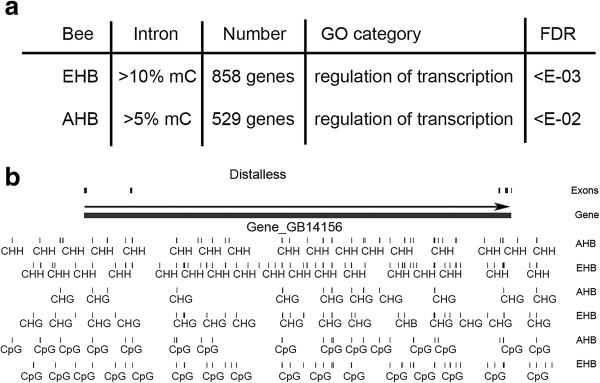
**CHH modifications are primarily in the introns of genes coding for transcription factors. a**, EHB and AHB intron methylation at CHH sites is enriched in the GO category “regulation of transcription” at the indicated FDR P values. There are 858 genes with >10% CHH modifications in EHB and 529 genes with >5% CHH modifications in AHB. Analysis with DAVID [32]. **b**, *Distalless* is a Hox gene and a transcription factor with high amounts of CHH modifications in the introns of both AHB and EHB.

We found that CHH modifications are highest in the introns of genes in the GO category “regulation of transcription” for both AHB and EHB (Figure 3a). This includes several Homeobox transcription factors, such as *Distalless* (Figure 3b) and *aristaless* (not shown). This is in contrast to genes with the highest CG methylation in exons, which were enriched in “housekeeping gene” GO categories, such as mitochondrial, ribosomal, and nucleotide-binding genes ( [6,8,9] and data not shown). As shown for *Distalless* (Figure 3b), introns in the “regulation of transcription” GO category often had a large amount of CG methylation in addition to the non-CG modifications. This again suggests that CG methylation and non-CG modifications are coordinately regulated.

### Non-CG modifications might regulate alternative mRNA splicing

**Figure 4:**
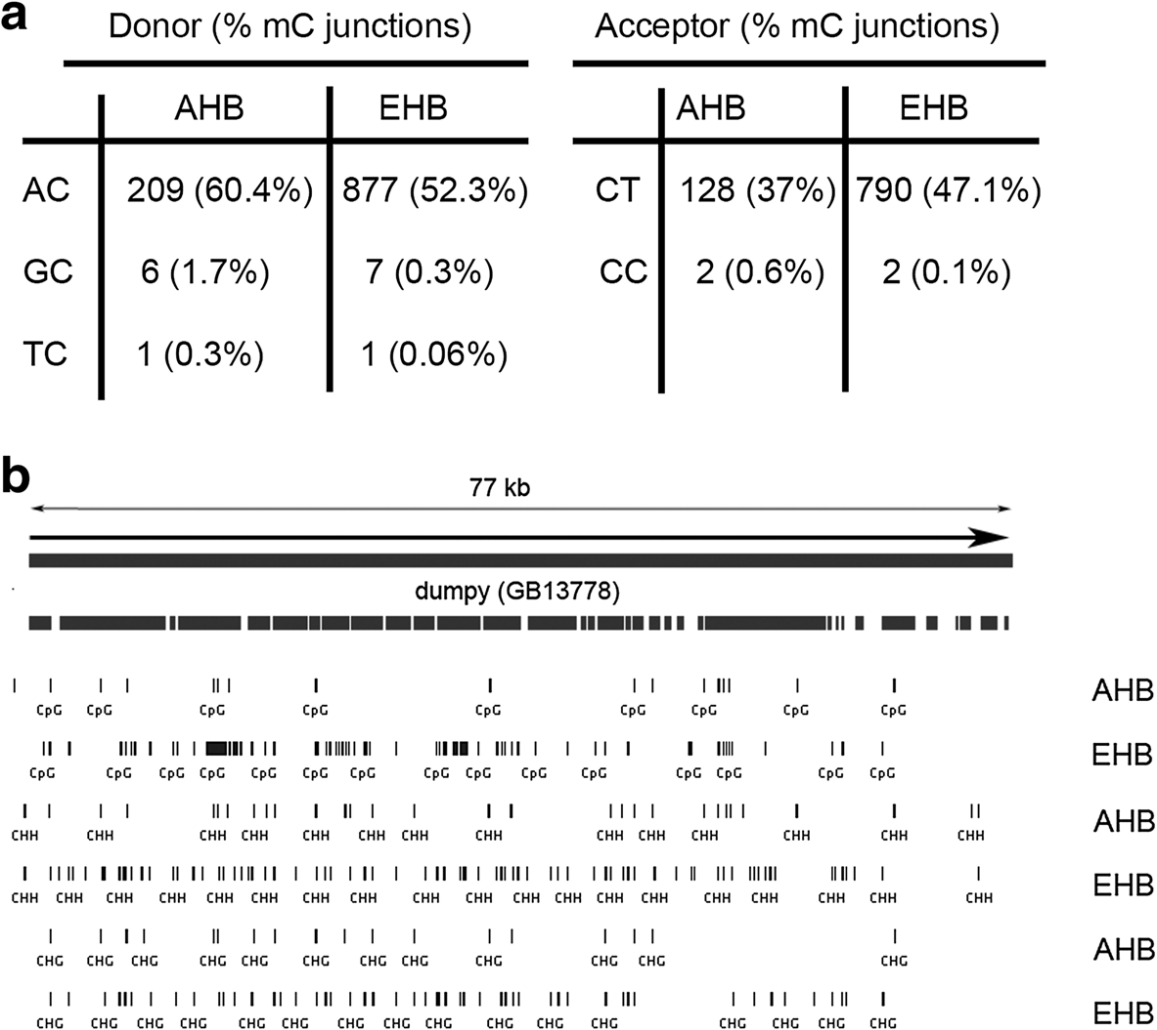
**Non-CpG methylation is enriched in splice junctions. a**, In AHB, over 97% of splice donors that are methylated on the template strand have the sequence GU (i.e., 209/216 have the template sequence A^m^C) and over 98% of splice acceptors that are methylated on the template strand have the sequence AG (i.e., 128/130 have the template sequence ^m^CT). Similarly in EHB, over 99% of splice donors that are methylated on the template strand have the sequence GU (i.e., 877/885 have the template sequence A^m^C) and over 99% of splice acceptors that are methylated on the template strand have the sequence AG (i.e., 790/792 have the template sequence ^m^CT). **b**, *dumpy* is a gene with the largest amount of splice junction methylation in both EHB (12 sites) and AHB (4 sites).

Consistent with the idea that DNA methylation might be involved in regulating mRNA splicing [8,9], we found that splice donors and acceptors were often encoded by DNA with non-CG modifications, such as either ^m^C or ^hm^C. In bees and other invertebrates, over 90% of splice donors have a G at the first position and a U at the second position (i.e., 5’-AC-3’ on the template DNA strand, where the C on the template strand can be methylated). We identified several hundred modified cytosines at splice donor and acceptor sites on the template strands (346 ^m^Cs in 321 genes in AHB, and 1677 ^m^Cs in 1312 genes in EHB) (Figure 4a).

Based on the above numbers, only ~0.66% of the ~56,000 splice junctions were methylated in AHB (346) and ~3.3% in EHB (1627) (Figure 4a). However, the distribution was clearly not random because pathway analyses show that genes with methylated splice sites were most enriched in the GO pathway “phosphoprotein” in both AHB and EHB (FDR < 10E-9 for both; Additional file 2: Table S5). Intriguingly, the GO pathway “alternative splicing” was also significantly enriched in both AHB and EHB (FDR < 0.05 for both; Additional file 2: Table S5). For example, the honey bee gene that was most heavily methylated at splice junctions is GB13778, the ortholog of *Drosophila dumpy*, whose protein products are involved in cell adhesion in *D. melanogaster*[33]; it has four methylated splice junctions in AHB and twelve methylated splice junctions in EHB at CHH and CHG sites (Figure 4b). Since *dumpy* has complex splicing programs in *Drosophila* (16 distinct mature spliced mRNAs are listed in FlyBase), and there are dozens of *dumpy* exons in honey bees, it is attractive to speculate that non-CG modifications at splice junctions in honey bees regulate alternative splicing at this locus and others as well.

In hESC lines, methylated non-CG sites are strongly conserved especially within the motif 5’-TA^m^CAG-3’ on the non-coding DNA strand at 3’ splice junctions [2]. While both hESCs and bees have non-CG modifications at splice junctions, bees differ from hESCs in several respects. In hESCs, methylation is symmetrical at CAG sites on both the template and non-template strand only at the 3’ splice junction, which is most frequently CAG. However in bees, methylation was primarily asymmetrical at CHH sites at the +1 position on the template strand encoding splice acceptors and the -1 position on the template strand at splice donors, and very few of the CHG sites in bees were symmetrically modified (Table 2). Another difference between hESC and bees is that the 3’ splice sites are predominantly methylated in humans but both 5’ and 3’ splice sites were modified in bees (Figure 4).

### Genes with more CHH modifications in EHB than AHB are enriched in behavioral response genes

Genes with significantly more CHH modifications in EHB than AHB were enriched in GO categories that are involved in neurological functions, such as “response to external stimulus”, “substrate specific channel activity”, “exocytosis”, and “neurotransmitter receptor activity” (Additional file 2: Table S6). These categories were highly significant even after correcting for the higher overall CHH modifications in EHB as well as multiple testing using the false discovery rate method (Additional file 2: Table S6) [20]. It is attractive to speculate that differential CHH modifications in introns might partially explain the striking behavioral differences between AHB and EHB, especially in aggression, but this is beyond the scope of the present study. Genetic studies suggest epigenetic regulation of aggression in vertebrates [34-38], and there are extensive aggression-related differences in brain gene expression between the aggressive AHB and the less-aggressive EHB [39]. Since there also are developmental differences between AHB and EHB, in addition to differences in aggressive behavior, these two types of differences would need to be teased apart in future studies.

### Gene expression positively correlates with both ^m^C and ^hm^C levels in the exons

We found a weak, but significant correlation between exon methylation and exon expression. This result was obtained for methylation detected by either bisulfite sequencing or Pvu-Seq (Additional file 1: Figure S9). This correlation was stronger for CG methylation than non-CG.

### Exons and Splice junctions with ^m^C or ^hm^C appear to affect alternative mRNA splicing

As stated above, several studies have suggested that DNA methylation might be involved in regulating mRNA splicing. To determine how our new ^hm^C finding might influence our understanding of the relationship between DNA methylation and mRNA splicing, we analyzed exons and splice junctions that differ in either ^m^C or ^hm^C between AHB and EHB and compared this with RNA-Seq data that we generated from AHB and EHB brains. We found several examples of differential ^m^C and ^hm^C associated with alternative mRNA splicing, as described below.

**Figure 5:**
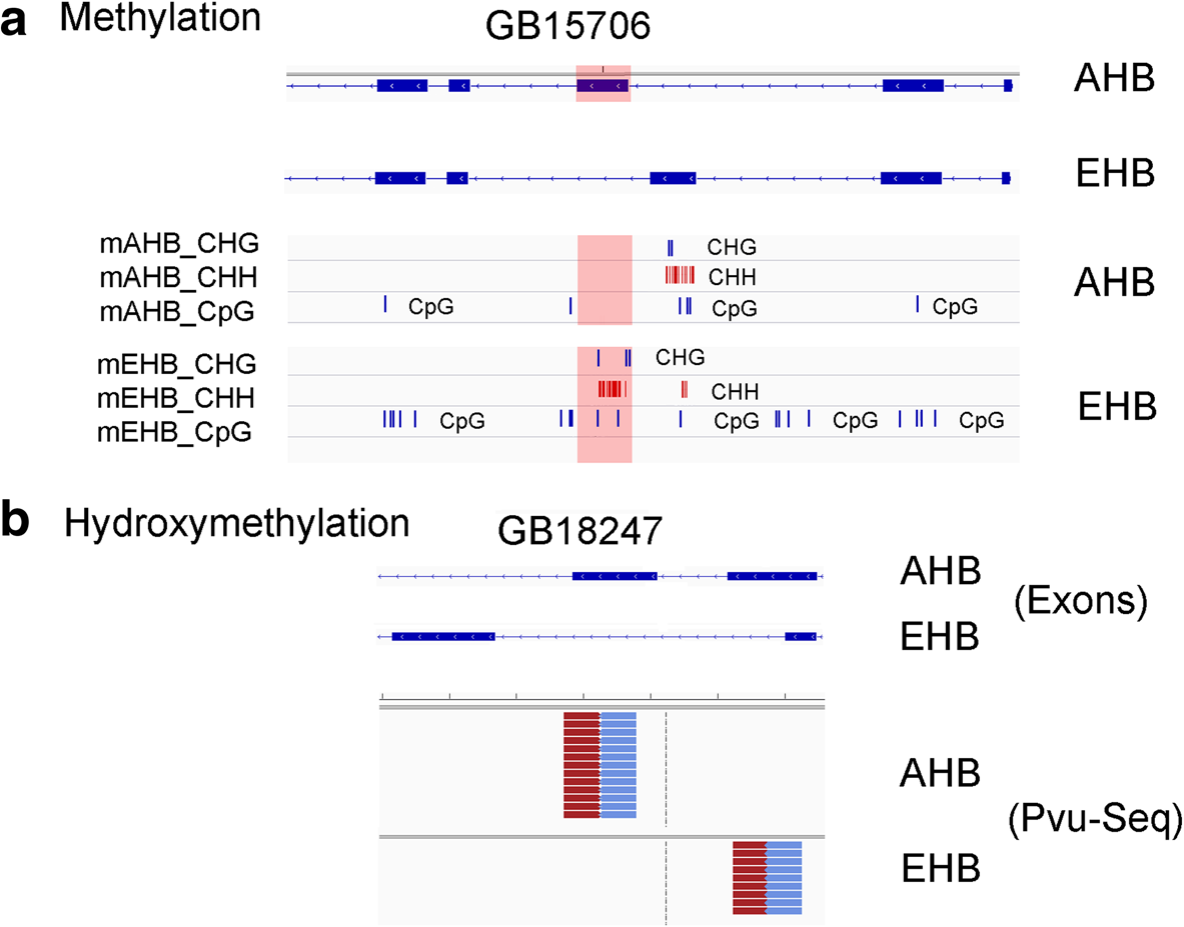
**Non-CG cytosine modifications might affect alternative mRNA splicing. a**, top blue lines indicated alternatively spliced exons in AHB and EHB. Underneath the exon sequences are the BS-Seq results showing the locations of CpG, CHG, and CHH ^m^C sites. Note that ^m^C in an exon correlates with the skipping of the exon by alternative splicing. For example, the gene GB15706, which encodes a homolog to Chromatin assembly factor 1 subunit A (CAF1A), has heavy CpG, CHH, and CHG methylation in a downstream exon that is skipped in AHB and present in EHB. Conversely, this gene has heavy cytosine methylation in the upstream exon in EHB that is skipped in EHB but present in AHB. Details of the RNA-seq analyses is in Additional file 1: Figure S7. **b**, Top blue lines indicated alternatively spliced exons in AHB and EHB, The red-and-blue stacked lines underneath the exons show Pvu-Seq analyses of AHB and EHB heads. Note that ^hm^C also correlates with alternative mRNA splicing, but in manners that differ from ^m^C (see text). Details of the RNA-seq analyses are given in Additional file 1: Figure S7.

Consistent with previous reports, we found several examples of differential CG methylation associated with alternative mRNA splicing (Figure 5a). Consistent with previously published examples [6], CG methylation in DNA encoding exons often correlated with that exon being skipped. For example, for gene GB15706, we observed that a heavily methylated exon in AHB was skipped, whereas an adjacent heavily methylated exon in EHB was skipped (see Additional file 1: Figure S8). We also report for the first time that non-CG modifications, such as ^hm^C, also showed a correlation with alternative splicing (Figure 5b). For example, the gene GB18247 had ^hm^C on an exon in AHB and that exon was retained in AHB, but that same exon did not have ^hm^C on it in EHB and that exon was skipped. In other words, at least for these examples, ^m^C on exons correlated with exon skipping, whereas ^hm^C on exons correlated with exon retention.

## Conclusions

Our findings underscore the diversity of DNA methylation mechanisms that exist. Non-CG modifications were only recently discovered in hESC and now we report them in the distantly related honey bee. We speculate that cytosine methylation at exons and splice junctions on the DNA might affect the mRNA splicing machinery. It is important to learn how these mechanisms work in relation to known regulators of splicing, such as histone acetylation [40], histone 3 lysine 4 methylation [41], histone 3 lysine 36 methylation [42], and histone 3 lysine 9 methylation [43,44].

Understanding how non-CG hydroxymethylation might affect alternative splicing is an exciting new area of research. Our data are consistent with a model in which DNA is methylated at CG sites by the maintenance DNA methyltransferase, Dnmt1, at exons, and at non-CG sites by the de novo DNA methyltransferase, Dnmt3, at introns. In contrast to Dnmt1, mammalian Dnmt3 is known to methylate both CG and non-CG sites in mammalian stem cells [31]. We speculate that the honey bee TET enzyme primarily recognizes the non-CG sites in the introns, thereby enriching DNA hydroxymethylation in the introns. We further speculate that the mRNA splicing machinery, as well as the histone modification machinery, distinguishes exons and introns by somehow recognizing the patterns of CG methylation in exons and non-CG hydroxymethylation in introns.

Our identification of GO categories related to protein phosphorylation that were enriched for genes with methylated splice junctions is consistent with a similar finding in a recent study of species-specific alternative exons [45]. The authors present evidence that argues that alternative splicing is used to alter protein phosphorylation, which can alter protein stability, subcellular localization, activity, and other properties [45]. Further research is needed to determine the mechanism by which splice junction methylation and hydroxymethylation affect mRNA splicing.

## Methods

### Sequencing

All sequencing was performed using Illumina Genome Analyzer GAIIx with a Paired End Cluster Generation Kit. Image analysis, base calling and sequence extraction was performed using standard Illumina Pipeline v1.6 software. We performed whole-genome shotgun sequencing of bisulfite modified DNA (BS-Seq). DNA sequencing (>20× coverage) also was performed to ensure that the C to U conversions were BS induced, rather than natural single nucleotide polymorphisms (SNPs; not presented here). BS-Seq was performed on Africanized honey bees (AHB) (*Apis mellifera scutellata*) and European honey bees (EHB) (a mixture of subspecies, primarily *A.m. ligustica*). The number of lanes sequenced was 11 (pair-end reads) for AHB and 8 for EHB, resulting in 240 million reads for AHB and 317 million reads for EHB. Reads were 76 base-pair long yielding a total of 18.2 giga bases and 24.1 giga bases for AHB and EHB respectively. We note that we used the Illumina Whole Genome Bisulfite Sequencing (WGBS) kit which first ligates non-methylated primers to the genomic DNA prior to bisulfite conversion. After bisulfite conversion, a second set of primers are used that only amplify the fully converted primers (e.g., [46]). We confirmed that only fully-converted primers were amplified in the BS-seq libraries (not shown).

### Bioinformatics analysis

The bioinformatics analysis was conducted by two different groups (D.M.R. and S.Z.), using different approaches and without sharing any processed data or results.

### Method 1 (BS-Miner)

The reference genome was Amel2, which is ~228 million bases long. At the time we performed our first bisulfite sequencing data analysis, there were only a few BS-Seq mappers available and some of them had a tendency to filter out non-CG methylation. For that reason, we decided to create our own unbiased analysis pipeline, which we called BS-Miner. It should be noted that nowadays many more options exist and those early mappers have been greatly improved, so there is no longer need to develop *ad hoc* methods. Read mapping and downstream analysis was done using BS-Miner. One of our main biological questions was whether non-CG methylation was present or not so we designed our pipeline using statistical methods well known in standard base calling algorithms. As a result, we obtain better sensitivity but only on 100%, 50% or 0% methylation levels (that is: methylated, hemi-methylated or no methylation). This sensitivity comes at a cost. As expected, the method does not detect methylation as a continuous range (from 0% to 100%) like other algorithms do. This design trade-off was aligned with our research hypothesis.

BS-Miner uses either BWA [47,48] or Bowtie [49] for read alignment. Both alignment programs are based on the Burrows-Wheeler [50] transformation and create SAM output format [51]. There are other tools and methods based on similar approaches [21,22,52]. In this case BWA was selected as the main mapping method in order to have better alignment near insertions and deletions. BS-Miner performs methylation calls by invoking Samtools [51], which uses a probabilistic model [53]. It must be noted that the BAQ model is explicitly disabled by BS-Miner, since some of its assumptions do not apply to methylation calls.

The BcfTools package was invoked to produce methylation calls in VCF format. BS-Miner was set to filter out low quality (Q < 20) methylation calls. After all mapping and filtering steps, the mean coverage was 20.8 and 27.4 for AHB and EHB respectively, which is > 2-fold more coverage than the previously most comprehensive bee study [5]. As a final step, BS-Miner performs several statistical analyses of the methylation results, including ranking of hypo-methylated and hyper-methylated genes by means of the Wilcoxon rank test and Fisher’s exact test. Multiple testing was corrected using False Discovery Rate methodology [20]. Some additional statistics were carried out using custom programs in R programming language [54] Further statistics from BS-Miner as well as additional analysis are available at http://www.mcb.mcgill.ca/~pcingola/bees/.

We also performed reanalysis of our data using Bowtie and a pipeline similar to the one shown in Krueger et al. [55]. Read trimming was performed using Trimmomatic [56]. Quality control was performed using FastQC (http://www.bioinformatics.babraham.ac.uk/projects/fastqc/) and in-house software. Performing reanalysis using different pipelines (BS-Miner and Bismark [21]) and different filtering strategies, we obtained consistent results. Removing duplicates using Bismark’s remove duplicates module [21], did not seem to change our results significantly.

### Method 2 (BSMap)

Using standard software, BSMap [23], we obtained results consistent with previous studies: methylation was primarily at CG dinucleotides in exons and very little non-CG methylation was present (Additional file 1: Figure S1a). There were 61,149,121 uniquely mapped cytosines (Cs) in EHB and 53,443,185 in AHB. More than 88% of EHB Cs and 83% of AHB Cs were covered by at least two sequencing reads (Additional file 1: Figure S2). However, when we included the reads that have DNA methylation in a non-CpG context, we found considerable amounts of CHH and CHG methylation (Additional file 1: Figure S1b). Bisulfite reads were mapped with BSMap to distinguish the methylated cytosine from unmethylated cytosines. The reads of bisulfite converted DNA were mapped to *Apis mellifera* genome assembly 4 after converting the Cs to Ts. Two mismatches were allowed for the alignment to be made. To reduce the potential erroneous cytosine methylation reads, the read for a cytosine was discarded if there was a mismatch event within the 2-bp surrounding context. There was also another “CHH-filter” which filtered out the entire read if the read contains three consecutive methylated CHH. The analyses are presented in two ways: with CHH-filter and without. For every gene, its 3k upstream region, 3k downstream region, and every exon or intron was divided into 30 bins and the ratio of the number of methylated cytosines over the number of all cytosines was plotted against the bin number. Bins were graphed from upstream (relative to the gene) to downstream.

### Comparison of methylation in AHB and EHB

The cytosine methylation in 3k upstream of all genes was averaged to calculate "upstream" methylation levels for every gene in both AHB and EHB; the cytosine methylation levels in gene body (or exons) were averaged to calculate the methylation level of gene body (or exons). To reduce bias from low mapping coverage, genes with less than 100 cytosines covered by BS-Seq reads, in gene body and 3k upstream, were excluded.

### Validating BS-Seq with MeDIP-Seq and HMeDIP-Seq analyses

Methylated DNA immunoprecipitation followed by sequencing (MeDIP-Seq or HMeDip-Seq) was performed for a total of 41.3 million 76 bp reads. Reads were aligned using BWA and SamTools. Peak-calling was performed using MACS 1.4 [57] beta version. Genomic DNA from 3 AHB and 3 EHB heads was sheared to 300-600 bp fragments, gel purified, immunoprecipitated with antibody, ligated to the library primers, amplified, and then sequenced. The mC antibodies were mouse monoclonal (Active Motif, Inc.) and the hmC antibodies were rabbit polyclonal (Active Motif, Inc.). Immunoprecipitation was with protein G beads (Active Motif, Inc.) following the manufacturer’s protocol. Additional file 1: Figure S4a shows the peak model based on the “forward reads” [those that align with the “Watson” (plus) strand] and the “reverse reads” [those that align with the “Crick” (minus) strand].

### Differential methylation analyses

Counts of methylation sites per gene, transcript, intron, splice sites and other regions of interest were calculated. Gene orthologs were calculated by InParanoid [58,59]. Enrichment was calculated using a greedy Wilcoxon rank sum method, RssGsc (http://www.rssgsc.sourceforge.net), which also performs multiple testing correction using false discovery rate. Fisher’s exact test was also applied as a secondary method, by setting a suitable threshold in the ranked list.

### Differencial SNP analysis

Counts of SNPs sites per gene, transcript, intron, splice sites and other regions of interest were calculated. Quantile normalization was applied and ranked genes analyzed for enrichment using the same methods as described in the previous section.

### Observed over expected (o/e) analysis

The observed over expected ratio is defined as the number of CG dinucleotides in the reference sequence, divided by the number expected under a uniform random distribution. For genes having multiple transcripts (which are a minority in amel2 reference genome), a weighted average was calculated according to each transcript’s length. This definition is consistent with [60].

### Accession numbers for data

The next-generation DNA sequencing experiments were done by the Applied Genomics Technology Core at Wayne State University. The next-generation DNA sequencing data (RNA-seq, Pvu-seq, MeDIP-seq, BS-seq, and HMeDIP-seq) was deposited into the GEO database according to the MINSEQE standards (Minimum Information about a high-throughput SeQuencing Experiment). The GEO database accession number is GSE50990. Gene Expression Omnibus (GEO; http://www.ncbi.nlm.nih.gov/projects/geo/).

### Animal use ethical use issues

Honey bees are not a regulated invertebrate. Therefore, no ethical use approval is necessary.

### GEO accession number

(GSE50990; http://www.ncbi.nlm.nih.gov/geo/query/acc.cgi?acc=GSE50990).

## Abbreviations

AHB: Africanized Honey Bee; EHB: European Honey Bee; Pvu-seq: PvuRts1I digestion of 5hmC sites followed by next generation DNA sequencing; BS-seq: Bisulfite treatment of DNA followed by next-generation DNA sequencing; ChIP-seq: Chromatin immunoprecipitation followed by next-generation DNA sequencing; MeDIP-seq: Immunoprecipitation of 5mC DNA with anti-5mC antibodies followed by next-generation DNA sequencing; HMeDIP-seq: Immunoprecipitation of 5hmC DNA with anti-5hmC antibodies followed by next-generation DNA sequencing; GO: Gene Ontology; DAVID: Database for Annotation, Visualization, and Integrated Discovery; hESC: Human embryonic stem cells; mC: 5-methylcytosine; hmC: 5-hydroxymethylcytosine; o/e: Observed over expected; VCF: Variable call format; SAM: Statistical analysis of microarrays; SNP: Single nucleotide polymorphism; Amel2: The reference genome for Apis mellifera (honey bee); MACS: Model based analysis for Chip-seq; BCF: Binary call format; CG: Cytosine-guanine dinucleotide; CHH: Cytosine followed by two nucleotides that are not guanine; CHG: Cytosine followed by one nucleotide that is not a guanine and then a guanine.

## Competing interests

The authors declare that they have no competing interests.

## Authors’ contributions

PC performed most of the bioinformatics analyses, XC and CC performed some of the methylation-related bioinformatic analyses, RK and MEH performed and analyzed the RNA-seq experiments, MC did most of the DNA sequencing, AS did some of the DNA sequencing, ABF helped with the Pvu-seq technique, SL and SZ supervised some of the bioinformatic analyses, YH did the CMS analyses, MDG helped edit the paper, GER helped write the paper and conceived of some of the experiments, DMR wrote most of the paper, conceived of some of the experiments, and supervised most of the genomic studies. All authors read and approved the final manuscript.

## Supplementary Material

Additional file 1 **Website of Data for Genome Browsers (Maintained by PC):**http://www.mcb.mcgill.ca/~pcingola/bees/**. Figure S1.** Coverage of BS-Seq data. **Figure S2.** BSMap analysis of BS-Seq data in AHB and EHB. **Figure S3.** Coverage by chromosome in AHB and EHB. **Figure S4.** Honey bees have hydroxymethylcytosine, as determined with dot blots. **Figure S5.** CpG methylation is highest in the exons of housekeeping genes. **Figure S6.** Differential CHH modifications is primarily in the introns of neuronal genes. **Figure S7.** RNA-Seq analysis of AHB and EHB heads compared with BS-Seq analyses. **Figure S8.** RNA-Seq analysis of AHB and EHB heads compared with Pvu-Seq analyses. Click here for file

Additional file 2: Table S1 BS-Miner analyses of data from Lyko et al. [6]. **Table S2.** BS-Map analysis of data from Lyko et al. [6]. **Table S3.** Cytosine DNA methylation in EHB and AHB in CG, CHG, and GHH genomic contexts. **Table S4.** Housekeeping genes have the most CpG DNA methylation. **Table S5.** AHB and EHB genes with methylated splice junctions. **Table S6.** Differential CHH modifications are in enriched in the introns of neuronal genes. Click here for file

## One sentence summary

Honey bee non-CG DNA hydroxymethylation is enriched in introns, which supplements previous findings that honey bee CG DNA methylation is enriched in exons.
