# Supplementary material for: Intronic Non-CG DNA hydroxymethylation and alternative mRNA splicing in honey bees
Source: BMC Genomics. 2013 Sep 30;14:666. doi: 10.1186/1471-2164-14-666 (PMC3850688; doi:10.1186/1471-2164-14-666)
Supplement: Additional file 1 — Website of Data for Genome Browsers (Maintained by PC):http://www.mcb.mcgill.ca/~pcingola/bees/. Figure S1. Coverage of BS-Seq data. Figure S2. BSMap analysis of BS-Seq data in AHB and EHB. Figure S3. Coverage by chromosome in AHB and EHB. Figure S4. Honey bees have hydroxymethylcytosine, as determined with dot blots. Figure S5. CpG methylation is highest in the exons of housekeeping genes. Figure S6. Differential CHH modifications is primarily in the introns of neuronal genes. Figure S7. RNA-Seq analysis of AHB and EHB heads compared with BS-Seq analyses. Figure S8. RNA-Seq analysis of AHB and EHB heads compared with Pvu-Seq analyses. [file 1471-2164-14-666-S1.doc]

**Figure S1.** Coverage of BS-Seq data. **A**, coverage of all cytosines in AHB (red) and EHB (blue). The mean, average, and standard deviation of coverage is shown. **B**, quality scores of all cytosines in AHB (red) and EHB (blue). A quality score of at least 20 reads was used in this paper (see Methods).

**Figure S2.** BSMap analysis of BS-Seq data in AHB and EHB. **A**, Cytosine methylation of filtered reads in AHB and EHB using BSMap. **B**, Cytosine methylation of unfiltered reads. Notice that all methylation levels are lower in the filtered reads compared with the unfiltered reads, and that CHH methylation is the most-affected by filtering.

**Figure S3.** Coverage by chromosome in AHB and EHB. **A**, coverage of plus strand (+ str), minus strand (-str), and both strands (DS) in EHB, and B, AHB by chromosome. Notice that the coverage of all sixteen chromosomes in bees is approximately equal.

**Figure S4.** Honey bees have hydroxymethylcytosine. **A**, Top row, example of a serial dilution of a standard oligo that has 5hmC replacing all of its cytosines. The antibody used was against cytosine-methylene sulfonate (CMS), which is the product of 5hmC after treatment with bisulfite. Bottom 4 rows, example of titration of a single head (AHB-Head and EHB-Head) or body (AHB-Body and EHB-Body). Notice that bodies have more hmC than heads. **B**, Quantitative levels of 5hmC DNA in the AHB and EHB heads and bodies. Each sample was analyzed six times and the mean values are shown. *, p value < 0.05, comparing EHB bodies to AHB bodies. NS, not significant.

**Figure S5.** CpG methylation is highest in the exons of housekeeping genes. **A**, scatter plot of cytosine methylation in AHB compared with EHB. The red line in the middle shows unity (AHB = EHB) and the parallel lines on either side of the unity line show 2-fold differences between AHB and EHB. In this plot, r = log2(m_ahb_+ α)/log(t_ahb_+α) and g = log2(m_ehb_+ α)/log(t_ehb_+α), where m_Xhb_ is the number of methylated bases and t_Xhb_ is the number of analyzed bases (i.e. total number of bases that could be methylated) and α = 1 (to prevent errors when there is no methylation). Both numbers (r and g) are then subtracted from their mean values (r_mean_ and g_mean_) so that the plots are centered at zero. **B**, density plot of A showing AHB (red) and EHB (green) densities. **C**, IGV browser snapshot of Ter94 ATP-binding housekeeping gene shows high levels of CpG methylation in both AHB and EHB.

**Figure S6.** Differential CHH methylation is primarily in the introns of neuronal genes. **A**, scatter plot of CHH methylation in introns in AHB versus EHB. The red lines and r and g are defined in Fig. S6. **B**, density plot of A showing AHB (red) and EHB (green) densities. **C**, Histogram Showing distribution of CHH sites in bee genes. o/e = observed over expected ratio. Notice that the CHH peak is unimodel rather than bimodal, as seen with exonic CG methylation (Fig. 2). **D**, Histogram showing the distribution of CHG sequences in bee genes. Notice that the peak is unimodel with an o/e peak at 0.5, suggesting the CHG methylation in genes is less frequent than expected. **E**, IGV browser snapshot of bifocal axon guidance gene showing high amounts of CHH methylation in the introns in both AHB and EHB.

**Figure S7.** RNA-Seq analysis of AHB and EHB heads compared with BS-Seq analyses. **A**, Top row, histogram of AHB RNA-Seq reads (from 1-20 reads per region). Underneath are individual RNA-Seq reads. **B**, Top row, histogram of EHB RNA-Seq reads (from 1-20 reads per region). Underneath are individual RNA-Seq reads. **C**, Top row, exons of GB15706 are shown. mC_peaks, shows MACS peak 9946 which is a mDIP-Seq peak from AHB heads. Underneath the MACS peak are shown the cytosine methylation at CHG, CHH, and CpG sites that were called by MoreBS. Notice that methylation of the first exon in EHB correlates with it being skipped in EHB and methylation of the second exon in AHB correlates with it being skipped in AHB.

**Figure S8.** RNA-Seq exon expression vs. methylation calls form BS-Seq (left column) and number of Pvu-Seq fragments (right column). Correlation is 0.12 and p-value is less than 10^-20^


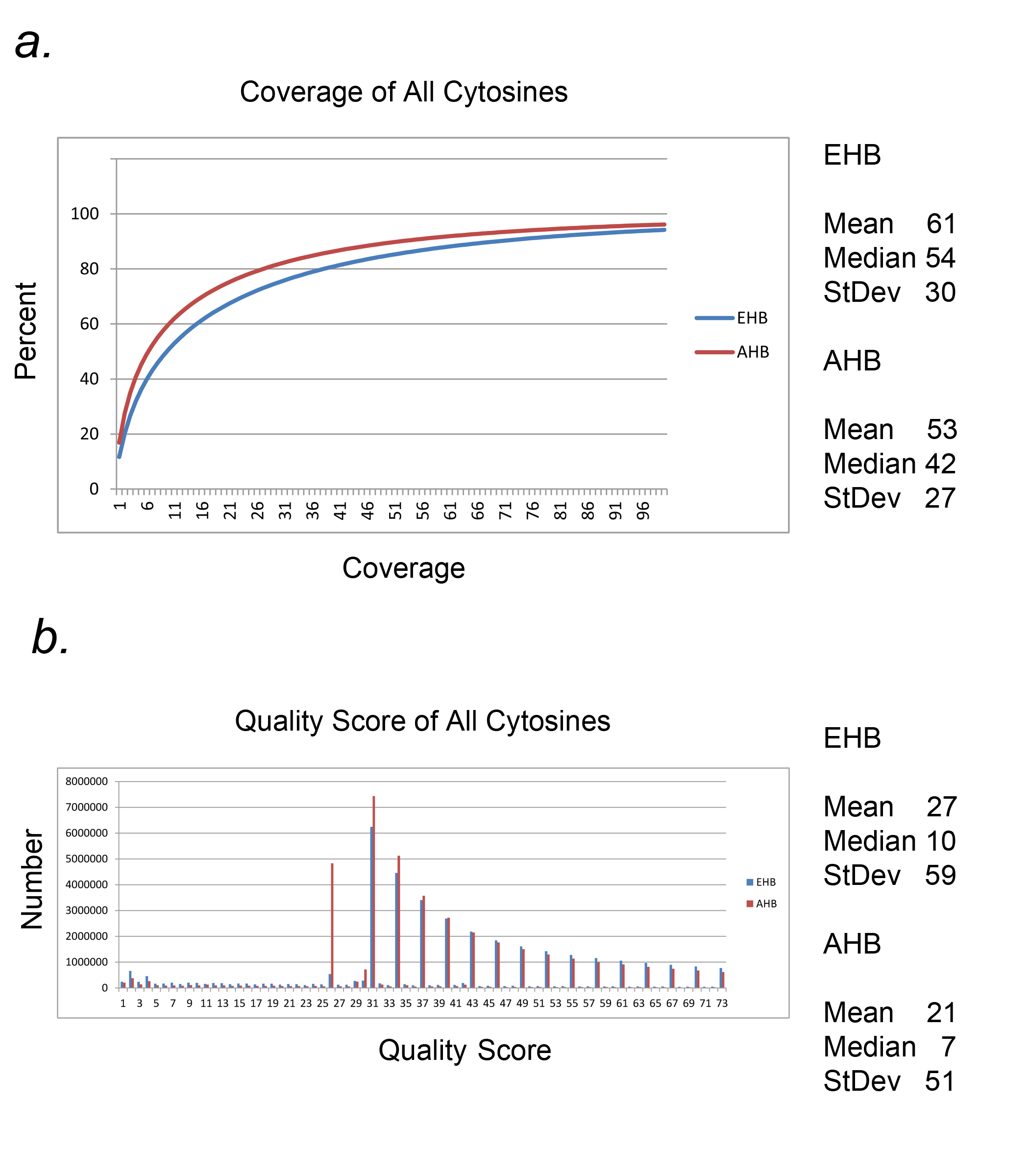


**Figure S1.** Coverage of BS-Seq data.


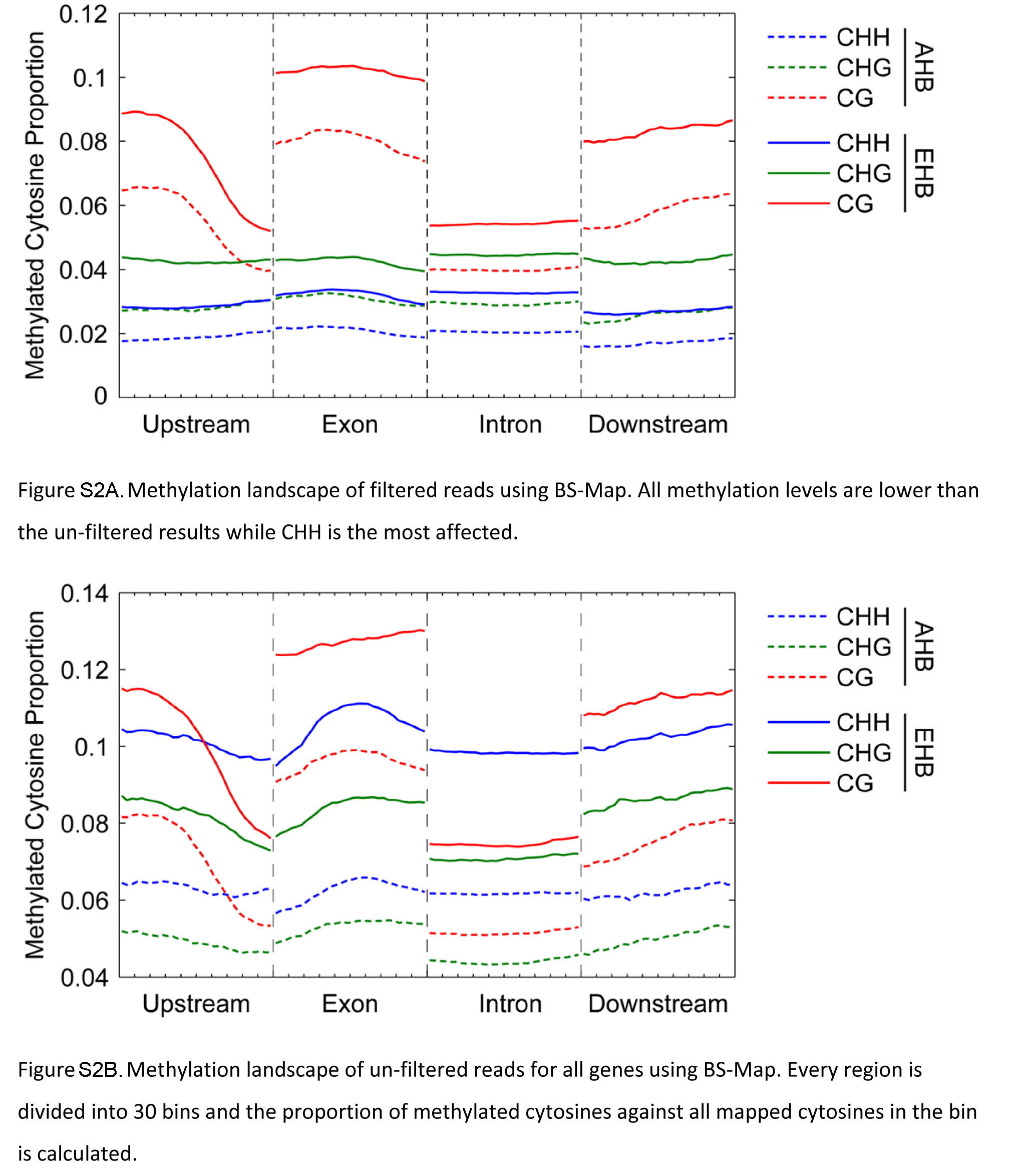


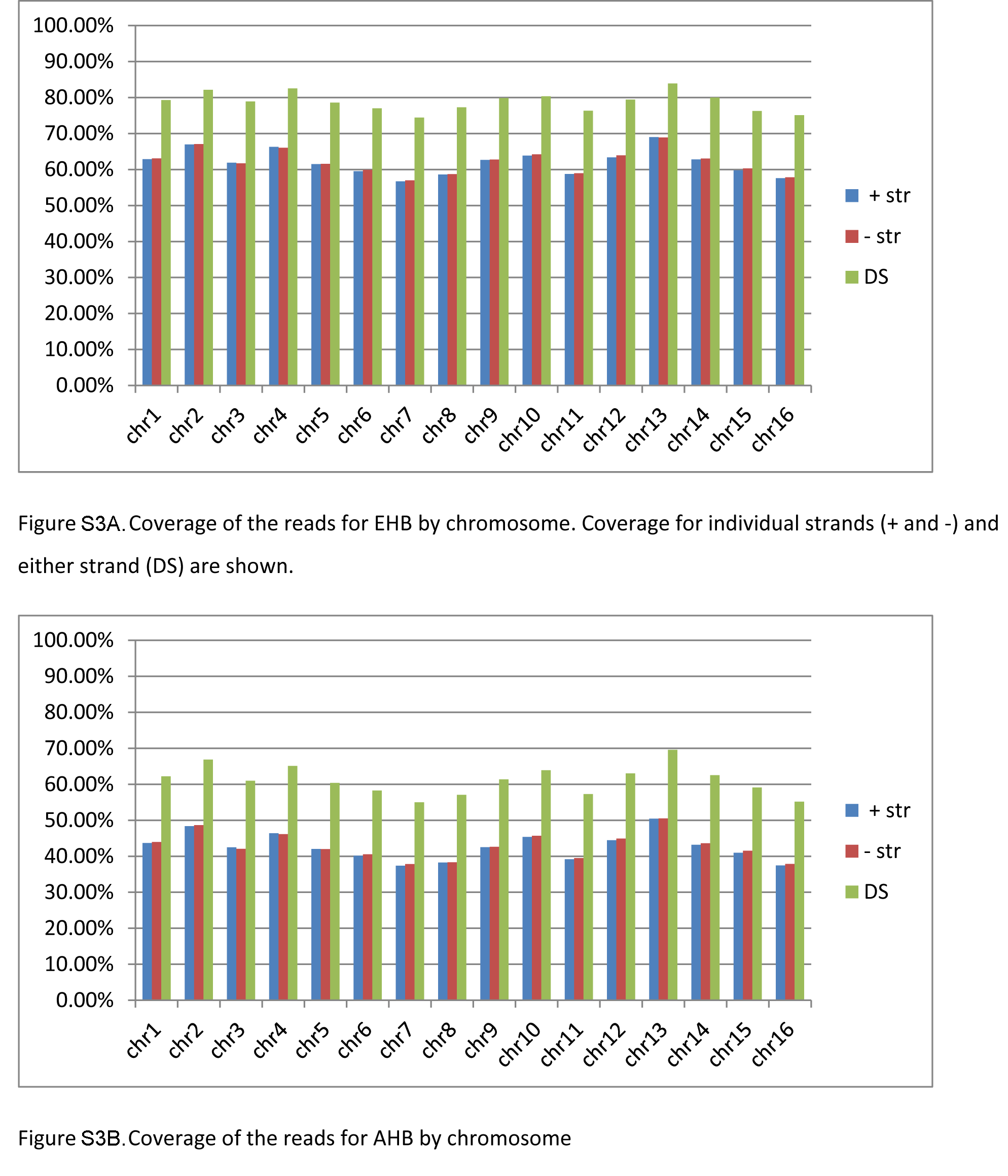


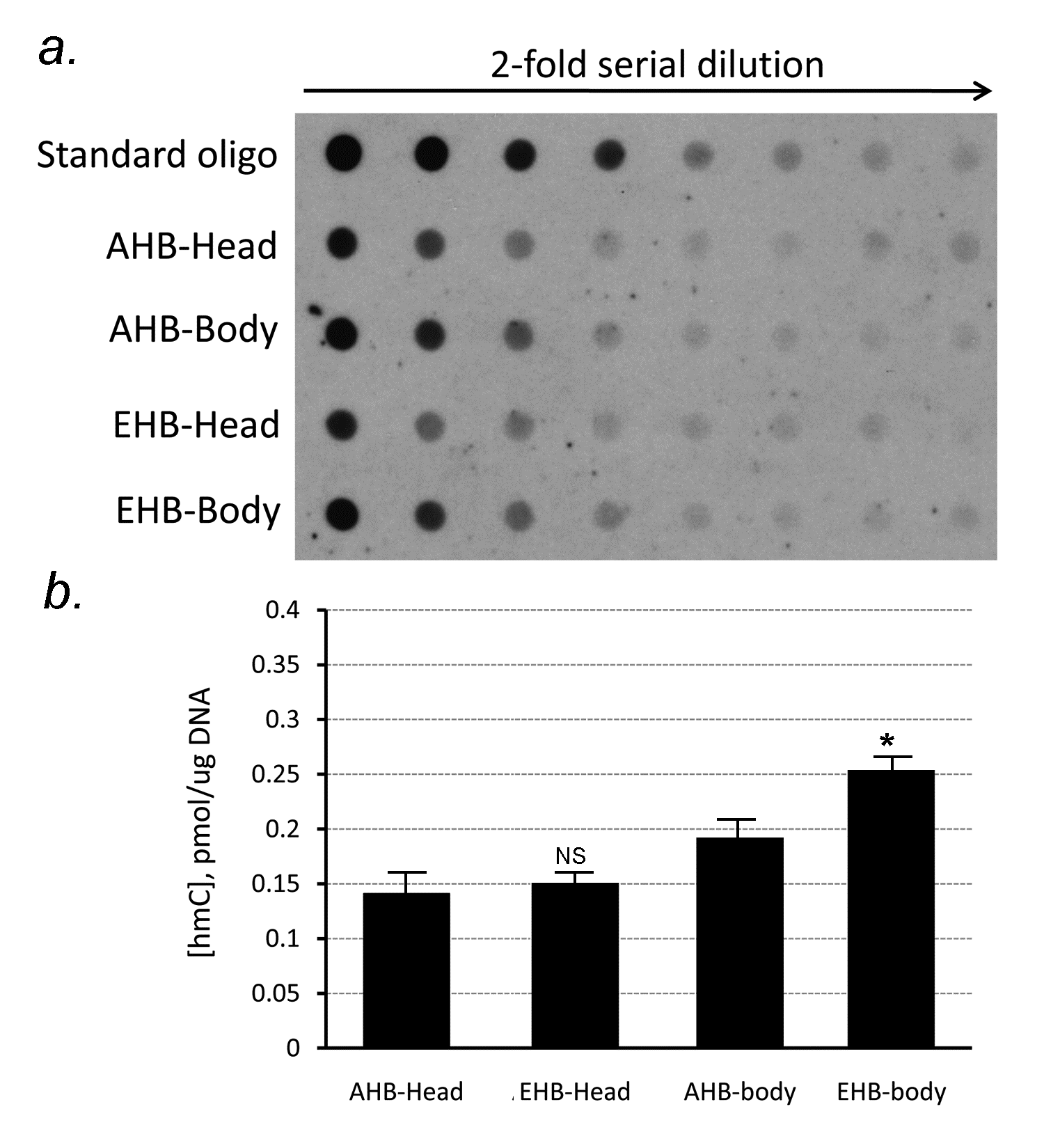


**Figure S4.** Honey bees have hydroxymethylcytosine.


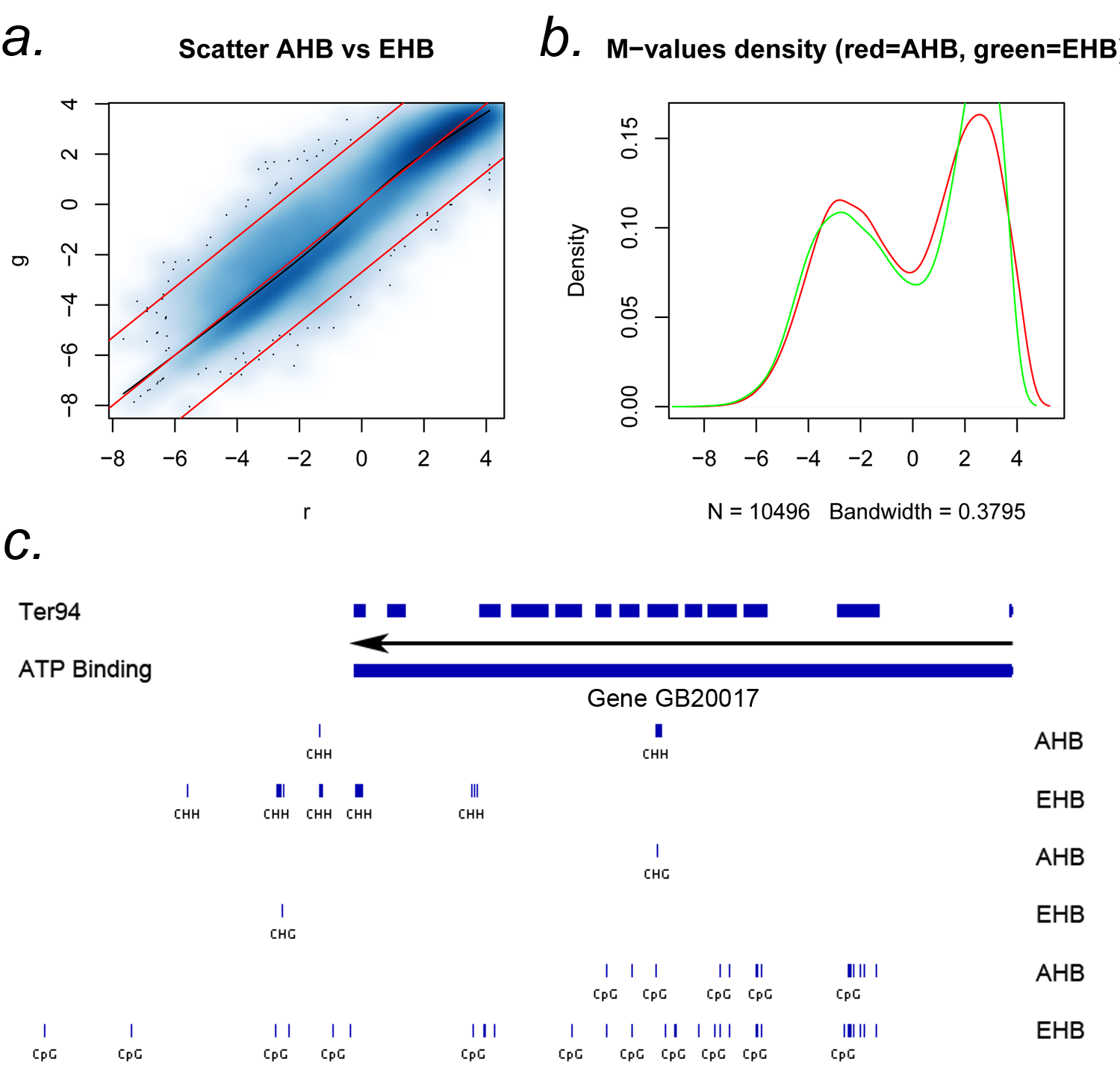


**Figure S5.** CpG methylation is highest in the exons of housekeeping genes.


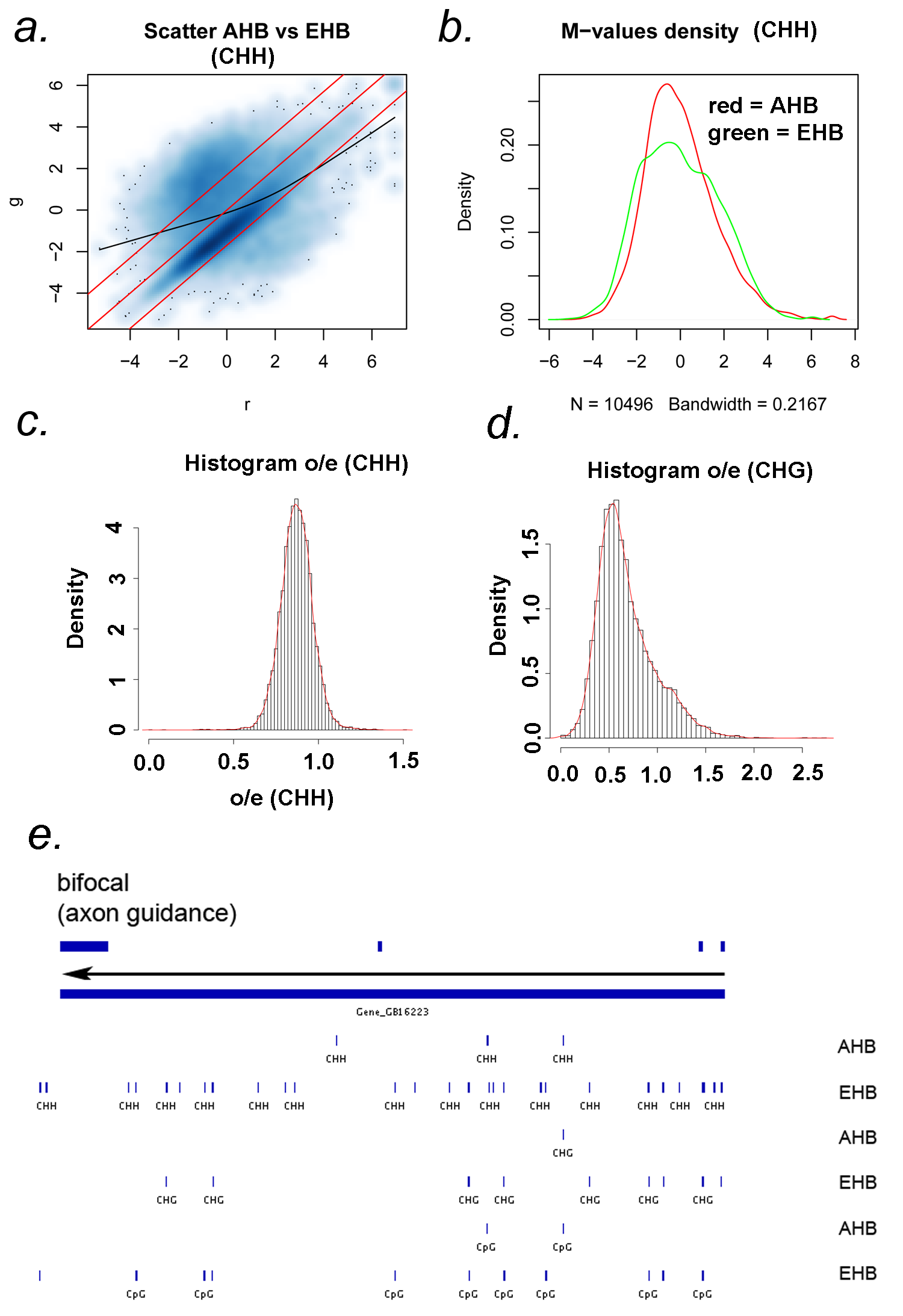


**Figure S6.** Differential CHH methylation is primarily in the introns of neuronal genes.


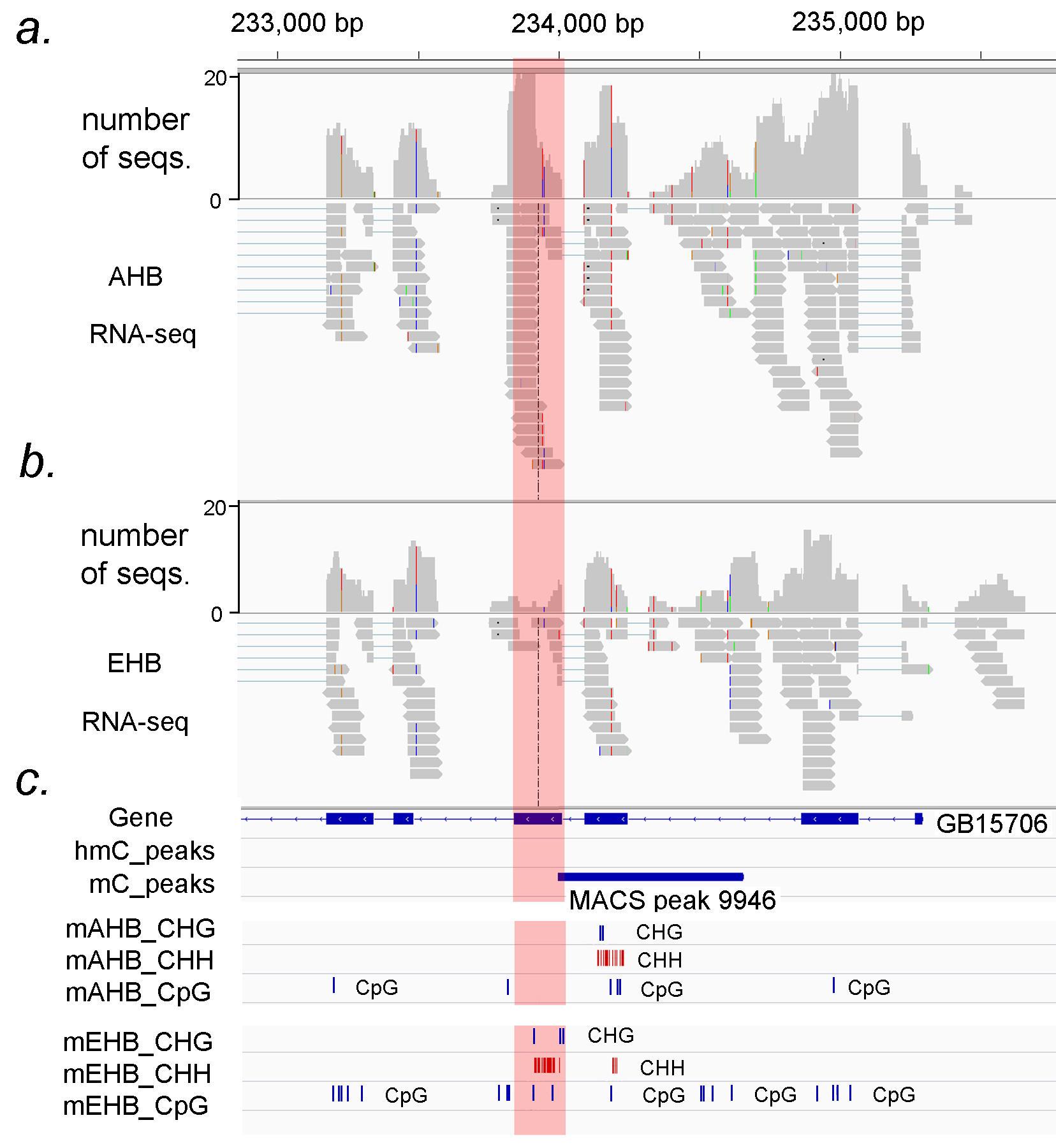


**Figure S7.** RNA-Seq analysis of AHB and EHB heads compared with BS-Seq analyses.

**
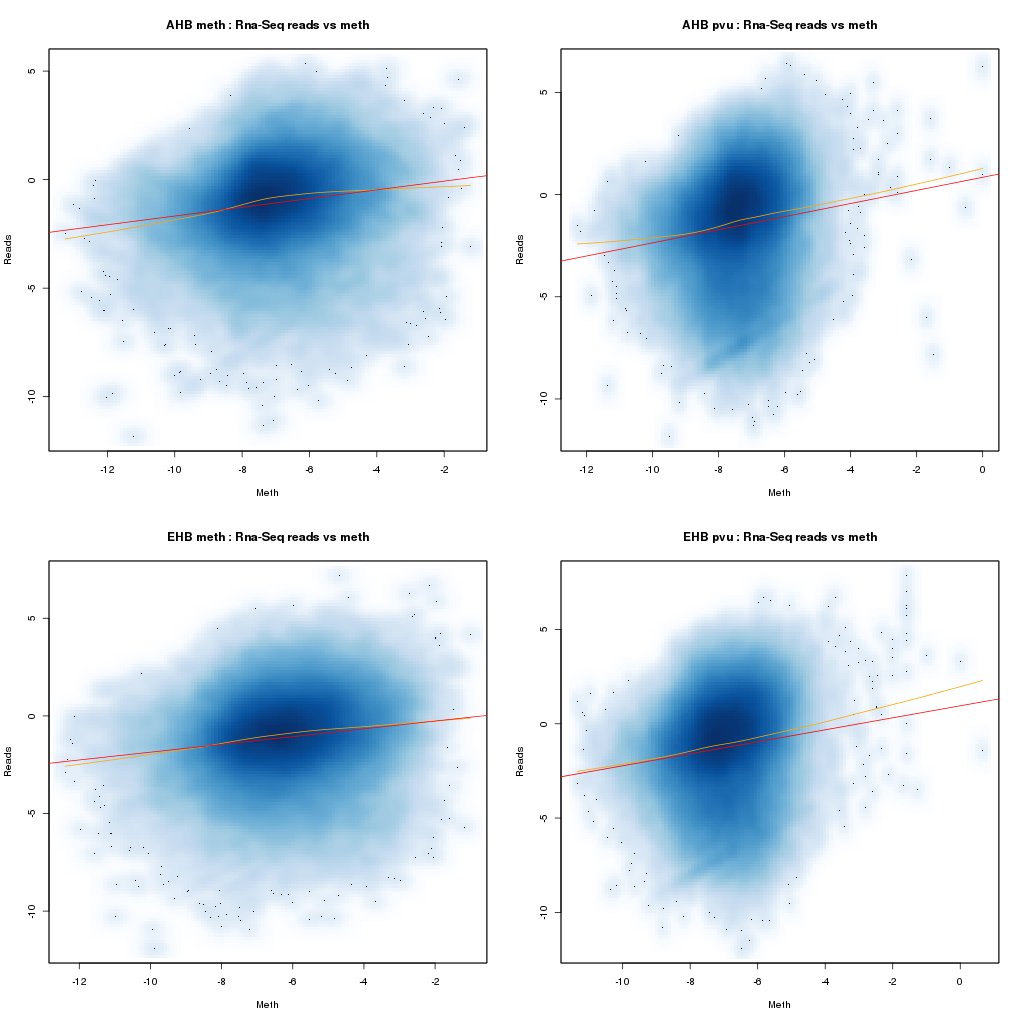
Figure S8.** RNA-Seq exon expression vs methylation calls form BS-Seq (left column) and number of Pvu-Seq fragments (right column). Correlation is 0.12 and p-value is less than 10^-20^
