# Supplementary material for: Intronic Non-CG DNA hydroxymethylation and alternative mRNA splicing in honey bees
Source: BMC Genomics. 2013 Sep 30;14:666. doi: 10.1186/1471-2164-14-666 (PMC3850688; doi:10.1186/1471-2164-14-666)
Supplement: Additional file 2: Table S1 — BS-Miner analyses of data from Lyko et al. [6]. Table S2. BS-Map analysis of data from Lyko et al. [6]. Table S3. Cytosine DNA methylation in EHB and AHB in CG, CHG, and GHH genomic contexts. Table S4. Housekeeping genes have the most CpG DNA methylation. Table S5. AHB and EHB genes with methylated splice junctions. Table S6. Differential CHH modifications are in enriched in the introns of neuronal genes. [file 1471-2164-14-666-S2.pdf]

Supplementary Tables

Table S1. BS-Miner analyses of data from Lyko *et al.* (2010).

Table S2. Bismark analysis of data from Lyko *et al.* (2010).

Table S3. Cytosine DNA methylation in EHB and AHB in CG, CHG, and GHH genomic contexts.

Table S4. Housekeeping genes have the most CpG DNA methylation.

Table S5. AHB and EHB genes with methylated splice junctions.

Table S6. Differential CHH methylation is enriched in the introns of neuronal genes.

Data	Ryszard					
Analysis	BS-Miner					
Sample	SRR039815					
Methylation type	CpG		CHH		CHG	
**Region**	**Count CpG**	**Percent**	**Count CHH**	**Percent**	**Count CHG**	**Percent**
DOWNSTREAM	97,300	29.05%	92,823	14.05%	3,561	15.81%
EXON	100,930	30.13%	17,150	2.60%	1,181	5.24%
INTERGENIC	18,652	5.57%	259,923	39.34%	8,009	35.56%
INTRAGENIC						
INTRON	24,967	7.45%	205,544	31.11%	6,390	28.37%
SPLICE_SITE_ACCEPTOR			217	0.03%	54	0.24%
SPLICE_SITE_DONOR	359	0.11%	245	0.04%	8	0.04%
UPSTREAM	92,741	27.69%	84,837	12.84%	3,321	14.74%
**Total**	334,949		660,739		22,524	
**Percent**	33%		65%		2%	

Table S1. BS-Miner analyses of data from Lyko *et al*. (2010).

**Data**	Ryszard					
**Analysis**	Bismark					
**Sample**	SRR039815					
**Methylation type**	CpG		CHH		CHG	
**Region**	**Count CpG**	**Percent**	**Count CHH**	**Percent**	**Count CHG**	**Percent**
DOWNSTREAM	125,846	23.30%	57,536	12.96%	8,737	12.09%
EXON	132,953	24.61%	27,882	6.28%	8,972	12.41%
INTERGENIC	78,899	14.61%	168,454	37.95%	24,219	33.50%
INTRAGENIC	20	0.00%	1	0%		
INTRON	80,428	14.89%	135,288	30.48%	20,735	28.68%
SPLICE_SITE_ACCEPTOR	1	0%	77	0.02%	90	0.12%
SPLICE_SITE_DONOR	395	0.07%	125	0.03%	28	0.04%
UPSTREAM	121,666	22.52%	54,486	12.28%	9,518	13.17%
**Total**	540,208		443,849		72,299	
**Percent**	51%		42%		7%	

Table S2. Bismark analysis of data from Lyko *et al*. (2010).

Type (alphabetical order) AHB_CpG	Analyzed	Methylated	Methylated %
Cds	1,273,706	40,288	3.163%
Downstream	2,086,041	37,798	1.812%
Exon	1,273,706	40,288	3.163%
Gene	7,105,672	62,011	0.873%
Intergenic	9,767,537	35,409	0.363%
Intron	5,887,038	22,463	0.382%
SpliceSiteAcceptor	14	1	7.143%
SpliceSiteDonor	2,257	81	3.589%
Transcript	7,806,591	64,990	0.833%
Upstream	2,294,855	38,169	1.663%

Type (alphabetical order) EHB_CpG	Analyzed	Methylated	Methylated %
Cds	1,318,533	82,558	6.261%
Downstream	2,255,737	89,512	3.968%
Exon	1,318,533	82,558	6.261%
Gene	7,555,968	149,371	1.977%
Intergenic	10,471,326	111,682	1.067%
Intron	6,293,493	68,460	1.088%
SpliceSiteAcceptor	18	1	5.556%
SpliceSiteDonor	2,504	215	8.586%
Transcript	8,297,751	158,204	1.907%
Upstream	2,468,142	87,960	3.564%

Table S3a.

Type (alphabetical order) AHB_CHH	Analyzed	Methylated	Methylated %
Cds	3,316,927	26,207	0.79%
Downstream	5,075,583	79,404	1.564%
Exon	3,316,927	26,207	0.79%
Gene	12,684,330	212,020	1.672%
Intergenic	16,317,387	325,139	1.993%
Intron	9,480,307	186,385	1.966%
SpliceSiteAcceptor	14,774	87	0.589%
SpliceSiteDonor	26,957	121	0.449%
Transcript	13,795,281	234,094	1.697%
Upstream	5,213,214	74,112	1.422%

Type (alphabetical order) EHB_CHH	Analyzed	Methylated	Methylated %
Cds	3,648,122	85,111	2.333%
Downstream	5,967,723	216,950	3.635%
Exon	3,648,122	85,111	2.333%
Gene	14,525,454	521,600	3.591%
Intergenic	19,011,050	773,971	4.071%
Intron	10,997,021	438,582	3.988%
SpliceSiteAcceptor	17,187	538	3.13%
SpliceSiteDonor	34,121	620	1.817%
Transcript	15,784,142	570,790	3.616%
Upstream	6,096,005	211,100	3.463%

Table S3b.

Type (alphabetical order) AHB_CHG	Analyzed	Methylated	Methylated %
Cds	1,002,706	3,493	0.348%
Downstream	1,119,219	4,559	0.407%
Exon	1,002,706	3,493	0.348%
Gene	3,188,727	11,619	0.364%
Intergenic	3,777,833	14,009	0.371%
Intron	2,225,532	8,217	0.369%
SpliceSiteAcceptor	16,858	43	0.255%
SpliceSiteDonor	4,407	15	0.34%
Transcript	3,470,589	12,577	0.362%
Upstream	1,189,498	4,526	0.38%

Type (alphabetical order) EHB_CHG	Analyzed	Methylated	Methylated %
Cds	1,059,063	11,898	1.123%
Downstream	1,242,115	16,772	1.35%
Exon	1,059,063	11,898	1.123%
Gene	3,419,998	37,559	1.098%
Intergenic	4,099,352	45,117	1.101%
Intron	2,401,495	25,966	1.081%
SpliceSiteAcceptor	18,474	257	1.391%
SpliceSiteDonor	5,072	57	1.124%
Transcript	3,718,898	40,695	1.094%
Upstream	1,308,187	16,511	1.262%

Table S3c. Distribution of CpG, CHH, and CHG DNA methylation.

EHB_>90%_CpG_514 genes								
GO	Count	%	List Total	Pop Hits	Pop Total	Fold Enrichment	PValue	FDR
mitochondrion	43	11.40584	164	571	4786	2.197663	5.85E-07	7.58E-04
ribosomal protein	17	4.509284	367	157	12980	3.82964	9.13E-06	0.011197
AHB_>90%_CpG_160 genes								
GO								
gtp-binding	15	7.8125	190	152	12980	6.74169	4.47E-08	5.14E-05
establishment of protein localization	17	8.854167	117	285	7937	4.046454	3.41E-06	0.005281

Table S4. Housekeeping genes have the most CpG DNA methylation. As found previously, the genes with high o/e ratios are primarily housekeeping genes (*1-3*) (Table S2). By 'housekeeping' genes, we mean genes that encode proteins with a higher degree of conservation than proteins encoded by non-methylated genes (*4*). Highly conserved genes are utilized in most tissues, whereas less conserved genes are typically expressed in specialized tissues. Examples are the genes coding for odorant-binding proteins or odorant receptors (*5*), which are not methylated (not shown, see also (*3*)).

Term_AHB	Count	%	PValue	List Total	Pop Hits	Pop Total	Fold Enrichment	FDR
phosphoprotein	45	21.32701	3.49E-13	206	815	12980	3.479064	4.27E-10
alternative splicing	25	11.84834	4.02E-05	206	616	12980	2.557212	0.049222

Table S5a. AHB genes with methylation splice junctions.

Term_EHB	Count	%	PValue	List Total	Pop Hits	Pop Total	Fold Enrichment	FDR
phosphoprotein	109	12.80846	9.10E-14	827	815	12980	2.099124	1.20E-10
GO:0001882~nucleoside binding	111	13.04348	1.35E-10	585	824	7918	1.823289	2.05E-07
nucleotide-binding	93	10.92832	3.20E-10	827	743	12980	1.964551	4.23E-07
GO:0001883~purine nucleoside binding	109	12.80846	3.64E-10	585	817	7918	1.805777	5.56E-07
GO:0030554~adenyl nucleotide binding	108	12.69095	5.55E-10	585	812	7918	1.800227	8.46E-07
atp-binding	78	9.165687	6.23E-10	827	585	12980	2.092705	8.25E-07
GO:0017076~purine nucleotide binding	123	14.45358	1.84E-09	585	989	7918	1.683327	2.80E-06
GO:0000166~nucleotide binding	142	16.68625	2.92E-09	585	1206	7918	1.593678	4.45E-06
GO:0005524~ATP binding	98	11.51586	9.75E-09	585	748	7918	1.773308	1.49E-05
GO:0032559~adenyl ribonucleotide binding	98	11.51586	1.13E-08	585	750	7918	1.768579	1.72E-05
GO:0032555~purine ribonucleotide binding	112	13.16099	5.03E-08	585	923	7918	1.642389	7.67E-05
GO:0032553~ribonucleotide binding	112	13.16099	5.03E-08	585	923	7918	1.642389	7.67E-05
cytoplasm	52	6.110458	1.71E-05	827	436	12980	1.871915	0.022614
alternative splicing	66	7.755582	3.52E-05	827	616	12980	1.681638	0.046545

Table S5b. EHB genes with methylated splice junctions.

chh_Intron M-EHB / M-AHB						
	Iteration	p-value	p-value adj	Latest result	Size	Description
	1	6.95E-17	5.19E-13	GO:0001071	395	nucleic acid binding transcription factor activity
	2	4.20E-23	2.34E-15	GO:0044459	369	plasma membrane part
	3	1.87E-27	7.80E-16	GO:0009605	363	response to external stimulus
	4	3.21E-30	9.98E-15	GO:0031012	37	extracellular matrix
	5	6.39E-33	1.48E-13	GO:0022838	197	substrate-specific channel activity
	6	2.54E-35	4.39E-12	GO:0005576	370	extracellular region
	7	2.01E-37	2.59E-10	GO:0005085	60	guanyl-nucleotide exchange factor activity
	8	1.65E-39	1.59E-08	GO:0006887	35	exocytosis
	9	1.31E-41	9.38E-07	GO:0030594	53	neurotransmitter receptor activity
	10	1.84E-43	9.84E-05	GO:0044449	32	contractile fiber part

Table S6. Differential CHH methylation is primarily in the introns of neuronal genes.
